# HSPB7 oppositely regulates human mesenchymal stromal cell-derived osteogenesis and adipogenesis

**DOI:** 10.1186/s13287-023-03361-0

**Published:** 2023-05-11

**Authors:** Shuang Zhang, Jeroen van de Peppel, Marijke Koedam, Johannes P. T. M. van Leeuwen, Bram C. J. van der Eerden

**Affiliations:** Laboratory for Calcium and Bone Metabolism, Department of Internal Medicine, Erasmus MC, Erasmus University Medical Center, Docter Molewaterplein 40, 3015 GD Rotterdam, The Netherlands

**Keywords:** Bone marrow-derived mesenchymal stromal cells, Osteogenic differentiation, Adipogenic differentiation, Lineage commitment, *HSPB7*, Activin A

## Abstract

**Background:**

Recent evidence suggests that accumulation of marrow adipose tissue induced by aberrant lineage allocation of bone marrow-derived mesenchymal stromal cells (BMSCs) contributes to the pathophysiologic processes of osteoporosis. Although master regulators of lineage commitment have been well documented, molecular switches between osteogenesis and adipogenesis are largely unknown.

**Methods:**

*HSPB7* gene expression during osteogenic and adipogenic differentiation of BMSCs was evaluated by qPCR and Western blot analyses. Lentiviral-mediated knockdown or overexpression of *HSPB7* and its deletion constructs were used to assess its function. The organization of cytoskeleton was examined by immunofluorescent staining. ALP activity, calcium assay, Alizarin Red S staining and Oil Red O staining were performed in vitro during osteoblast or adipocyte differentiation. SB431542 and Activin A antibody were used to identify the mechanism of Activin A in the regulation of osteogenic differentiation in BMSCs.

**Results:**

In this study, we identified *HSPB7* capable of oppositely regulating osteogenic and adipogenic differentiation of BMSCs. *HSPB7* silencing promoted adipogenesis while reducing osteogenic differentiation and mineralization. Conversely, overexpression of *HSPB7* strongly enhanced osteogenesis, but no effect was observed on adipogenic differentiation. Deletion of the *N*-terminal or *C*-terminal domain of *HSPB7* led to decreased osteoblastic potency and mineralization. Mechanistically, our data showed that Activin A is a downstream target participating in *HSPB7* knockdown-mediated osteogenic inhibition.

**Conclusions:**

Our findings suggest that *HSPB7* plays a positive role in driving osteoblastic differentiation, and with the capability in maintaining the osteo-adipogenesis balance. It holds great promise as a potential therapeutic target in the treatment of bone metabolic diseases.

**Supplementary Information:**

The online version contains supplementary material available at 10.1186/s13287-023-03361-0.

## Background

Osteoporosis is the most common skeletal disorder characterized by low mineral density and bone structure deterioration, leading to increased fracture risk in the ageing population. In addition to the well-established concept that osteoporosis is caused by the imbalance between osteoblasts and osteoclasts [1, 2], growing evidence suggests the involvement of aberrant lineage allocation of bone marrow-derived mesenchymal stromal cells (BMSCs) [3, 4]. BMSCs are multipotent cells with the ability of self-renewal and multiple lineage differentiation including osteoblasts and adipocytes [5, 6]. However, this lineage commitment process is generally regarded to be inversely correlated, as osteogenic differentiation of MSCs requires suppression of adipogenesis, while increased marrow adipogenic differentiation takes place at the expense of osteoblast formation [7, 8]. Consistent with these findings, human studies have proposed that accumulated marrow adipose tissue is correlated with osteoporosis and increased fracture risk, particularly in the context of obesity and aging, further implicating the impairment of lineage allocation [9–13].

Cell fate decision and commitment of MSCs are strictly orchestrated by a variety of physical factors and molecular signals [14]. Cell shape and cytoskeletal (re)arrangement also contributes to lineage specification, as MSCs confined to a ‘star’ shape showed increased osteogenesis whereas ‘flower’ patterned confinement led to enhanced adipogenesis [15]. Transcription factors, such as RUNX2 and Osterix (SP7), are classically considered as the master regulators of osteogenesis [16], while PPARγ and CEBPα/β/δ play essential roles toward adipogenesis [17]. WNT and Hedgehog are well-known signals to stimulate osteogenic and antagonize adipogenic differentiation [18]. Some crucial domains within specific proteins could further prime MSCs toward specific lineages and mediate the response of MSCs to certain lineage-specific stimulators [19]. Identifying molecular switches is important to dictate the reciprocal relationship between osteoblast and adipocytes during fate determination and the ultimate control of osteo-adipogenic balance.

Small heat shock proteins (sHSPs), characterized by low molecular weight and highly conserved *C*-terminal domains (α-crystallin) were originally discovered as molecular chaperons against protein misfolding in pathological conditions. Over the past decade, numerous studies have demonstrated that sHSPs physically interact with different types of transcription factors as well as intrinsic and extrinsic signals, indicating a possible role in stem cell behavior. Evidence suggests that sHSPs are involved in bone metabolism through specific pathways regulating cell differentiation [20], growth factor secretion [21] and calcium deposition [22]. Being the most widely studied sHSP in osteoblasts, HSPB1 is critical for vascular endothelial growth factors (VEGF) release induced by transforming growth factor (TGF)-β and fibroblast growth factor (FGF)-2 [21, 23]. The non-phosphorylated version of *HSPB7* could decrease the expression of osteocalcin, further enhancing mineralization in osteoblasts [14]. Dysregulation of HSPB8 has been associated with impaired osteogenic differentiation of dental pulp stem cells, further establishing the important role of sHSPs during osteoblast differentiation [22].

*HSPB7* is one of the least studied members in sHSP family and is highly expressed in heart and skeletal muscle [24]. In this study, we demonstrated that *HSPB7* capable of oppositely regulating osteogenic and adipogenic differentiation, indicating it to be a molecular switch mediating lineage allocation of BMSCs.

## Methods

### Cell culture and differentiation

Human bone marrow-derived mesenchymal stromal cells (BMSCs, Lonza, Basel, Switzerland) were cultured as previously described [25]. Briefly, BMSCs were maintained in alpha minimum essential medium (α-MEM, Gibco, Paisley, United Kingdom) supplemented with 10% heat-inactivated fetal calf serum. Following two days of attachment, osteogenic induction was initiated using 100 nM dexamethasone and 10 mM *β*-glycerophosphate. For adipogenic induction, BMSCs were treated with 0.1 μM dexamethasone, 60 μM indomethacin, and 0.5 mM 3-isobutyl-1-methylxanthine. Cells at passage 7 were used in all experiments and media was refreshed every 3 or 4 days. To block Activin A activity, SB431542 (Sigma-Aldrich, Zwijndrecht, the Netherlands) or Activin A neutralizing antibody (R&D Systems, Minneapolis, Minnesota, United States) was added during cell refreshment.

### Generation of constructs and lentivirus-mediated knockdown and overexpression

As previously described [26], the constructs of short hairpin RNA (shRNA) targeting *HSPB7* and the nontargeting shRNA with a scrambled sequence serving as negative control were purchased from TRC-1.0 library (Sigma-Aldrich, Zwijndrecht, the Netherlands; Additional file 1: Table S1). To obtain overexpression, full-length human *HSPB7* cDNA (Horizon Discovery, Waterbeach, United Kingdom) containing a His-tag stop codon was cloned into a pEntr-TOPO vector and transferred by Gateway recombination into a pLenti6.3/V5–DEST vector (Gateway Vector Kits, Life Technologies Europe B.V., the Netherlands). *HSPB7* deletion constructs were generated using Q5^®^ Site-Directed Mutagenesis Kit (New England Biolabs, Massachusetts, Unite States) according to the manufacturer’s instructions. Following proofreading PCR (Primers were shown in Additional file 1: Table S2), the amplified product was added to a Kinase–Ligase–DpnI enzyme mix for 5 min enabling room temperature (RT) circularization and template removal. Subsequent products were transformed into *E. coli* and plasmid DNA isolation was performed after culture. All constructs were verified by Sanger sequencing.

Lentivirus was produced by transient transfection into 293FT cells using a standard calcium phosphate precipitation method with the addition of 1 μg/ml polybrene (Sigma-Aldrich, Zwijndrecht, the Netherlands) as described previously [27]. After 48 h, supernatants containing lentivirus were harvested and used immediately for BMSC transduction (24 h after attachment). One day later, medium was replaced with differentiation induction medium, and cells were cultured until further analysis.

### Alkaline phosphatase (ALP) activity and mineralization assays

ALP activity was determined using *p*-nitrophenyl phosphate (*p*NPP) as a phosphatase substrate which turns yellow when dephosphorylated to *p-*Nitrophenol (*p*NP) by ALP. As previously described [25], cell extracts were harvested at different time points using PBS containing 0.1% triton X-100. The conversion step from *p*NPP to *p*NP was performed for 10 min at 37 °C. ALP activity was quantified by measuring the absorbance at 405 nm and adjusted to the total protein content. Total protein concentration was determined using a BCA protein assay kit (Thermo fisher scientific, Waltham, Massachusetts, United States) following manufacturer’s instruction.

For mineralization, cell lysates and the remaining plates were incubated overnight with 0.24 M HCl at 4 °C. Calcium content was determined in a colorimetric way using a combination of 1 M ethanolamine buffer (pH 10.6) with 0.35 mM 0-cresolphthalein in a 1:1 ratio. Total calcium content was calculated by combining calcium in cell lysates and calcium left behind in the plates. Alizarin Red S staining was performed as described previously [27]. Briefly, cells were fixed with 70% ethanol and stained for 15 min with Alizarin Red S solution at RT (pH 4.2, Sigma-Aldrich, St. Louis, Missouri, United States).

### Oil red O staining

After 14 days of adipogenic differentiation, BMSCs were washed twice with PBS, fixed with 10% formalin, and subsequently stained with Oil Red O solution (Sigma-Aldrich, St. Louis, Missouri, United States). Cell number was determined by DAPI staining and pictures were taken by a Zeiss Axiovert 200MOT microscope (Zeiss, Sliedrecht, The Netherlands). Normalized absorbance was calculated using raw absorbance divided by cell count.

### Cell viability assay

Cell viability was determined using Cell Counting Kit-8 (CCK-8) assays. BMSCs in the presence or absence of induction were incubated with CCK-8 reagent (Sigma-Aldrich, St. Louis, MO, USA) for 2 h in an incubator according to the manufacturer’s manual. The conversion of the tetrazolium salt WST-8 to formazan was measured at 450 nm.

### RNA isolation and quantification of mRNA expression

RNA isolation, cDNA synthesis and real-time PCR reactions were performed as described before [25]. Oligonucleotide primer pairs were designed to be either on exon boundaries or spanning at least one intron (Additional file 1: Table S3). Gene expression was normalized to the expression of the housekeeping gene *36B4*, using the equation. 2^^−(Ct gene of interest – Ct housekeeping gene)^.

### Immunostaining

Immunostaining was performed as previously described [27, 28]. Briefly, BMSCs were fixed with 4% PFA for 5 min at RT and washed with PBS. Immunostaining was performed after permeabilization with 0.1% Triton X-100 (Sigma-Aldrich, St. Louis, Missouri, United States) in PBS and blocking for 30 min in 1% bovine serum albumin at RT simultaneously. Cells were incubated with α-Tubulin antibody (1:100; Cell signaling #2125S, The Netherlands) or *HSPB7* antibody (1:100; Novus Biologicals NBP1-84334, Abingdon, United Kingdom) overnight at 4 °C. The next day, cells were incubated with Alexa Flour 488 donkey anti-rabbit (1:200; Abcam #150073, Cambridge, United Kingdom) secondary antibody for 1 h at RT, followed by the addition of rhodamine-conjugated phalloidin (1:100, Thermo Fisher Scientific #10063052, Massachusetts, United States) for 1 h at RT. After 10 min incubation with DAPI, images were taken with a confocal laser scanning microscope (Leica Microsystems, Wetzlar, Germany).

### Western blot

Western blot analysis was performed as described [28]. Total protein was collected in RIPA lysis buffer (Thermo Fisher Scientific, Massachusetts, United States). Equal amounts of protein per sample were loaded and separated by SDS-PAGE (Bio-Rad Laboratories B.V., Veenendaal, The Netherlands) and transferred onto a polyvinylidene difluoride membrane (Amersham™ Hybond^®^ Western blotting membranes, Sigma-Aldrich, Zwijndrecht, the Netherlands). Each membrane was blocked with 5% non-fat milk in Tris-buffered saline containing 0.1% Tween-20 (TBS-T) at RT for 1 h before blotting with primary antibodies directed against *HSPB7* (1:1,000; Novus Biologicals NBP1-69,072, Abingdon, United Kingdom), β-Actin (1:1,000, Cell signaling #4970, The Netherlands), PPARγ (1:1,000; Cell signaling #2435, The Netherlands), C/EBPα (1:1,000; Cell signaling #8178, The Netherlands), Perilipin (1:1,000, Cell signaling #9349, The Netherlands), FABP4 (1:1,000, Cell signaling #2120, The Netherlands), Fatty acid synthase (1:1,000; Cell signaling #3180, The Netherlands), Acetyl-CoA carboxylase (1:1,000, Cell signaling #3676, The Netherlands) at 4 °C overnight. After three washes in TBS-T, the membrane was incubated with anti-rabbit antibody (1:2,000; Cell signaling #7074, The Netherlands) conjugated with horse radish peroxidase for 1 h at RT. The proteins of interest were detected with Gel Doc XR System (Bio-Rad Laboratories B.V., Veenendaal, The Netherlands) using the Clarity™ Western ECL Substrate (Bio-Rad Laboratories B.V., Veenendaal, The Netherlands) and were semi-quantified using Image Lab software (Bio-Rad Laboratories B.V., Veenendaal, The Netherlands).

### Statistical analysis

Data were displayed as means ± SE of representative experiments. All experiments were performed at least two times. Statistical analysis was performed using GraphPad Prism 9. Significance was calculated using the Student’s *t*-test, and one-way or two-way ANOVA after post-hoc testing.

## Results

### HSPB7 is upregulated during osteogenic differentiation and downregulated during early adiopogenesis

**Fig. 1:**
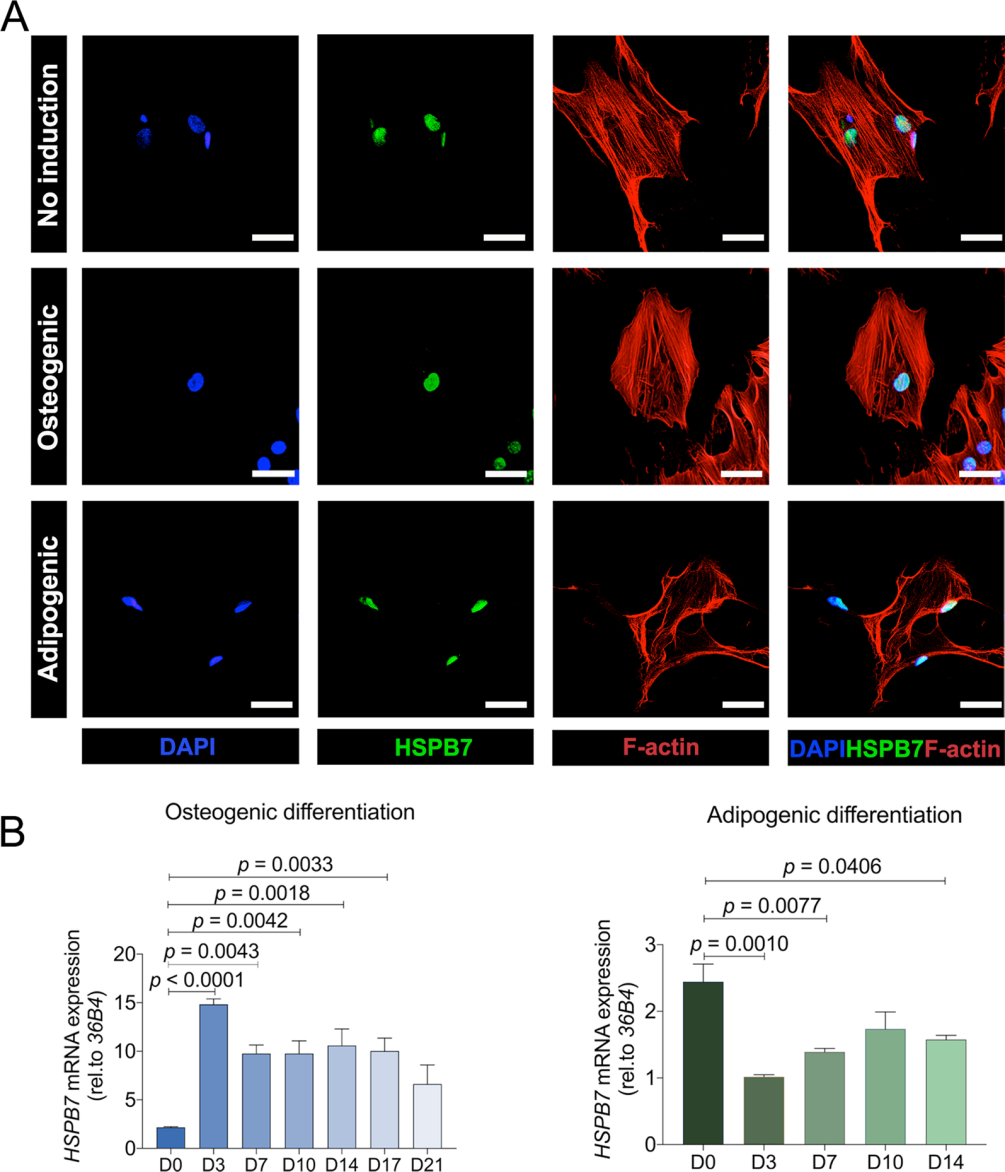
*HSPB7* expression is upregulated during osteogenic differentiation and downregulated during early adipogenic differentiation. **A** Cellular localization of HSPB7 in BMSCs with or without induction at day 3. **B** Relative mRNA expression levels of *HSPB7* in BMSCs were assessed by qRT-PCR over 3 weeks. BMSC cultured in osteogenic condition was indicated with blue bars, and adipogenic condition was indicated with pink bars. Data are presented as means ± SEM and analyzed by one-way ANOVA (*n* = 2 per group) followed by post-hoc testing. Scale bars: 200 μm

sHSPs are widely known to be distributed throughout cellular compartments but currently there is no indication of *HSPB7* protein localization in BMSCs. Immunofluorescent detection reveals that *HSPB7* is exclusively localized in the nucleoplasm in the presence or absence of induction (Fig. 1A). To determine the role of *HSPB7* during BMSC differentiation, we measured the expression level of *HSPB7* under osteogenic and adipogenic induction of BMSCs (Fig. 1B). In comparison with undifferentiation stage (day 0), *HSPB7* expression is dramatically increased during osteogenesis, peaking at 3 days of differentiation. On the other hand, the expression of *HSPB7* is decreased during early days of adipogenic differentiation, showing an opposite trend to that of osteogenic inductions. These data led us to further investigate *HSPB7* as a candidate gene influencing BMSC differentiation.

### Silencing HSPB7 in BMSCs inhibits osteogenic differentiation and mineralization

**Fig. 2:**
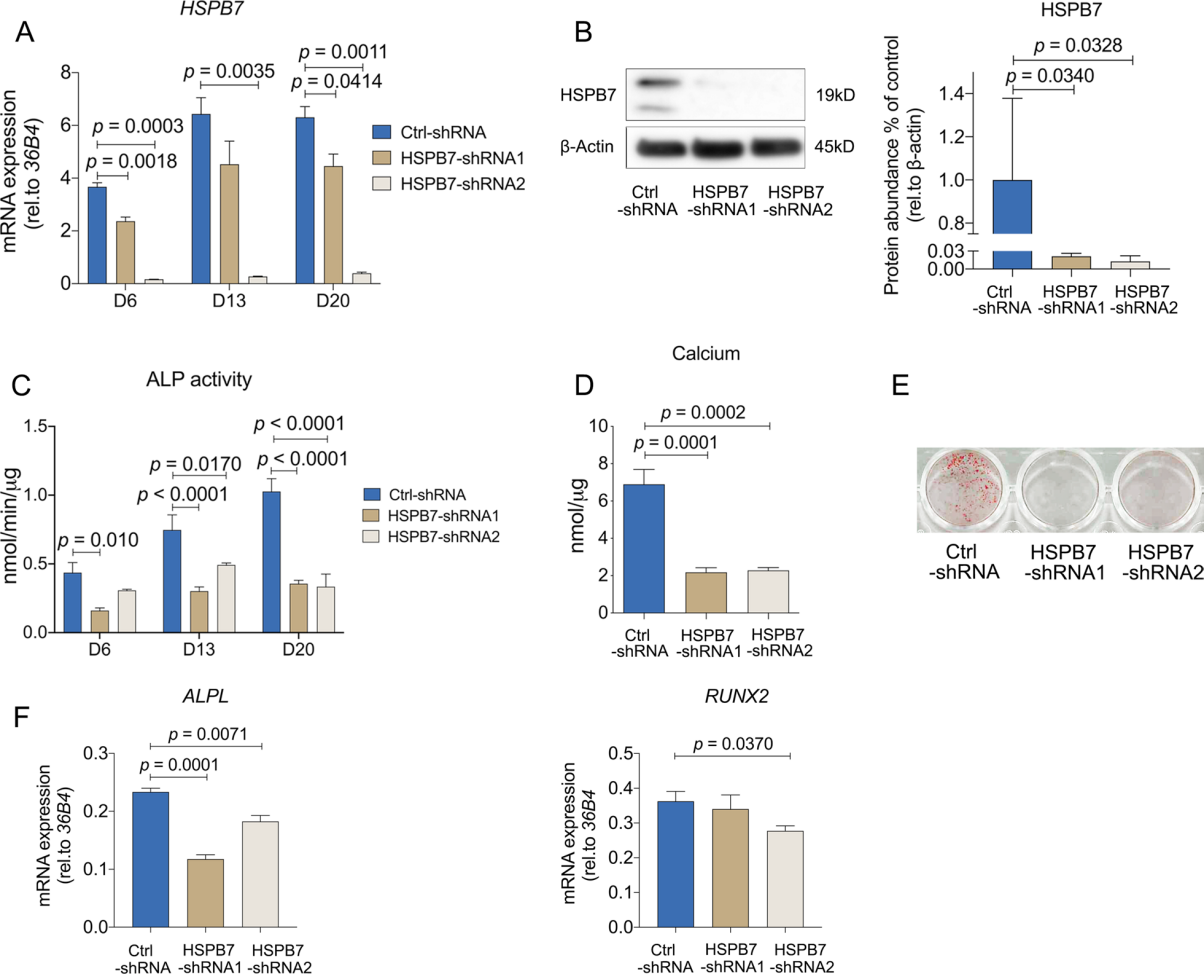
*HSPB7* silencing in BMSCs inhibits osteogenic differentiation and mineralization. **A**–**B** *HSPB7* silencing was assessed by quantification of HSPB7 mRNA expression by qRT-PCR at different time points (**A**) and Western blot (**B**) on day 3 under osteogenic induction. Full-length blots are presented in Additional file 7: Fig. S6. **C** ALP activity was evaluated at different time points under osteogenic induction. **D**–**E** Osteoblast mineralization was assessed by calcium deposition assay (**D**) and Alizarin Red S staining (**E**) after three weeks osteogenic induction. **F** osteogenesis-related genes were evaluated on day 6. Data are presented as means ± SEM and analyzed by one-way ANOVA (**B**, **D** and **F,** *n* = 3–4 per group) or two-way ANOVA (**A**, *n* = 4 per group) followed by post-hoc testing

To delineate the role of *HSPB7* in osteoblast fate decision, we first performed *HSPB7* gene silencing in BMSCs. Efficient knockdown of *HSPB7* was evaluated by gene expression analysis and Western blot. Figure 2A demonstrates that *HSPB7* mRNA was inhibited by two different shRNAs, and these effects were further confirmed at the protein level (Fig. 2B). Cell viability indicated by CCK-8 experiment was decreased following *HSPB7* silencing (Additional file 2: Fig. S1A), suggesting a potential effect on cell proliferation.

Next, we examined the consequence of *HSPB7* silencing on classical biochemical markers during osteogenic differentiation and mineralization of BMSCs. BMSCs showed significantly less ALP activity at different time points after treatment with osteogenic induction media (Fig. 2C). Remarkably, mineralized nodule formation was completely abolished as shown by calcium deposition assay and Alizarin Red S staining (Fig. 2D–E). When assessing osteoblast marker genes, reduced levels of *RUNX2* and *ALPL* were detected 6 days after *HSPB7* silencing (Fig. 2F). Taken together, these findings indicate that *HSPB7* inhibition impaired osteogenic differentiation.

### HSPB7 silencing in BMSCs affects cytoskeleton reorganization

**Fig. 3:**
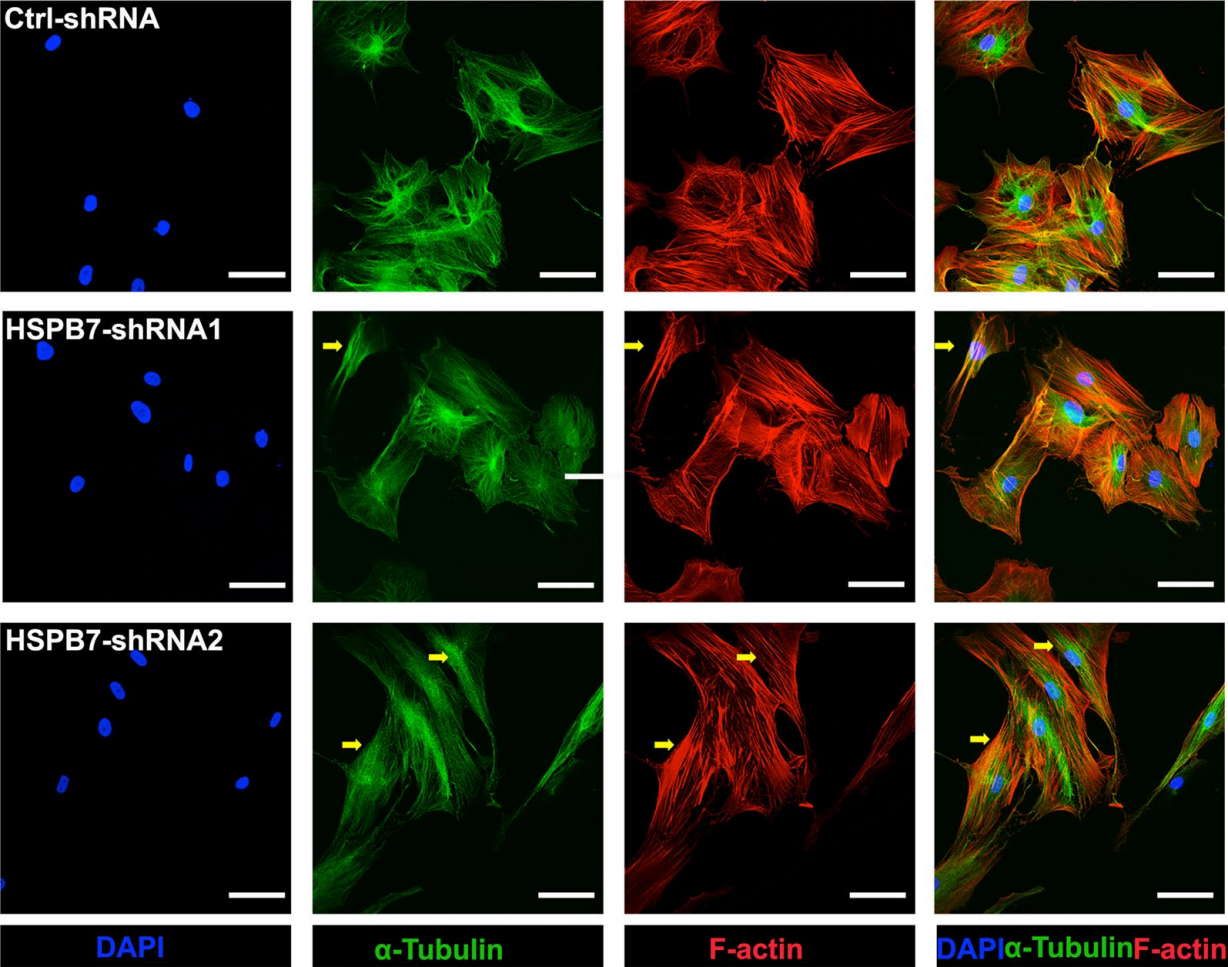
*HSPB7* silencing in BMSCs affects cytoskeleton organization. A Representative images of immunostainings against F-actin (phalloidin-rhodamine), α-tubulin (Alexa Fluor 488) and Nuclei (DAPI) at day 14 following osteogenic induction. Arrows indicate spindle-shaped fibroblast-like cells. Scale bars: 200 μm

One of the most important features of sHSPs is the ability to interact with cellular components of the cytoskeleton, and this interaction protects the cytoskeleton from injury in a stressful environment [29]. Given that BMSCs change from a fibroblast-like phenotype to a spherical shape during osteoblastic differentiation, and end up as mature osteoblasts (or osteocytes), we hypothesized that *HSPB7* participates in the rearrangement process of the cytoskeleton during osteogenic differentiation and mineralization. Therefore, we monitored the dynamic changes of microfilament and microtubule organization by immunostaining at multiple time points. BMSCs cultured in osteogenic media exhibited progressive morphological changes as indicated from day 3 to day 20, as thick actin patterns were recorded running across the entire cytoplasm toward the outermost parts of the cells during osteogenic differentiation (day 14 in Fig. 3, days 3, 7 and 20 in Additional file 3: Fig. S2A–C). However, there was no substantial reorganization regarding the microtubule structure.

In contrast to the cuboidal phenotype of the osteoblasts in the control situation, *HSPB7* silenced BMSCs (shRNA2) displayed a spindle-shape fibroblast-like morphology on day 14, with parallel actin filaments observed in the cytoplasm (Fig. 3). Remarkably, microtubule networks ran through the entire cell body in parallel rather than radiating from a perinuclear location (Fig. 3), as shown at different time points from day 3 to day 20 during osteogenic differentiation (Fig. 3, Additional file 3: Fig. S2A–C). Together, these data suggest that *HSPB7* silencing during osteogenic differentiation leads to dynamic cytoskeletal changes influencing cell morphology.

### Silencing HSPB7 in BMSCs enhances adipogenesis

**Fig. 4:**
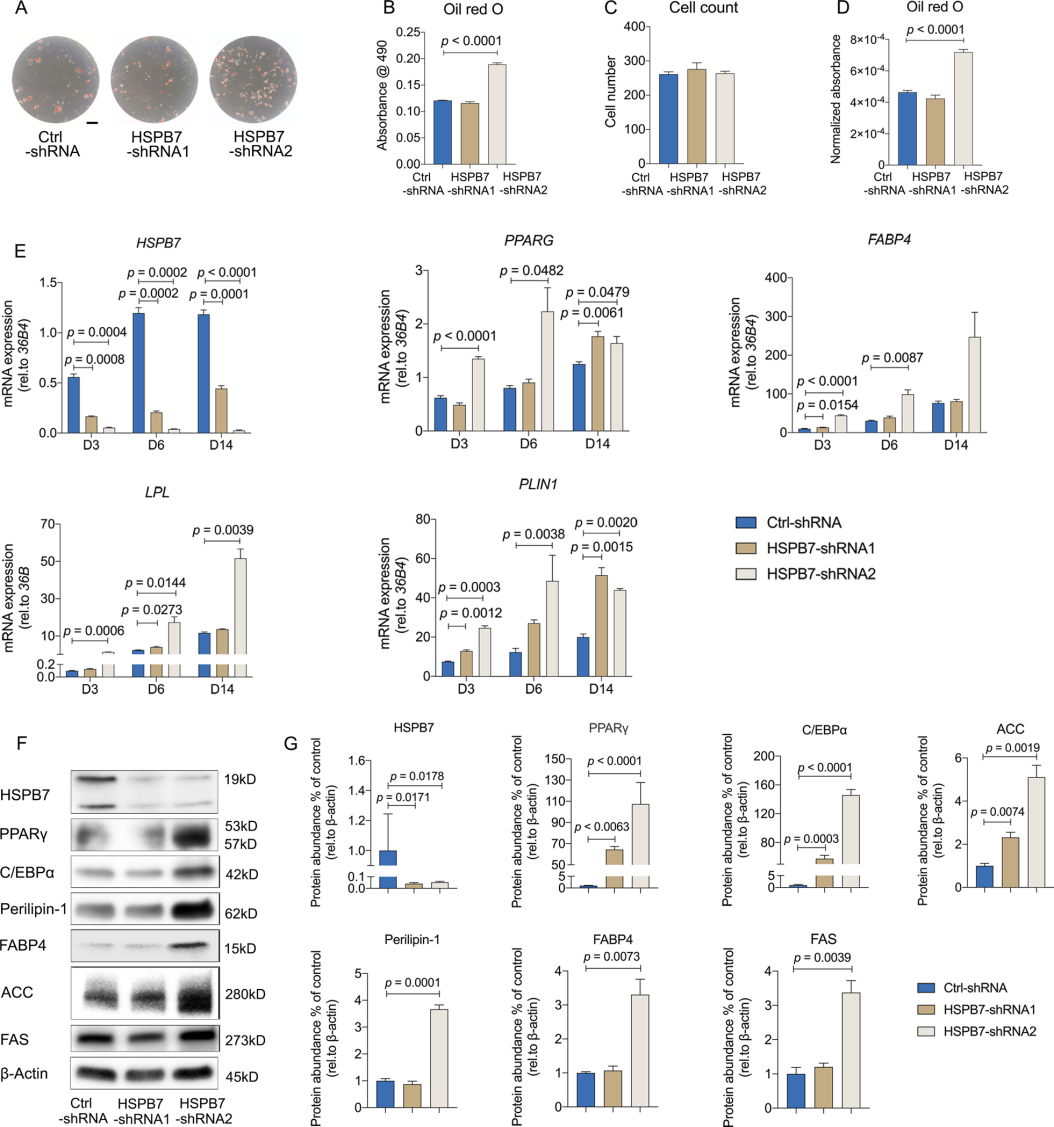
*HSPB7* silencing in BMSCs enhances adipogenesis. **A**–**D** Representative images (**A**) and quantitative data (**B**) of Oil Red O staining performed after 14 days with adipogenic induction. Total cell number was determined by DAPI staining (**C**). Quantitative data of Oil Red O staining were adjusted by cell number (**D**). **E** The expression of adipogenic markers was evaluated by qRT-PCR at indicated time points. **F**–**G** Representative images (**F**) and quantitative data (**G**) of adipogenic genes were assessed on day 7 following adipogenic induction by Western blot. Full-length blots are presented in Additional file 7: Fig. S6. Abbreviations: Peroxisome proliferator-activated receptor gamma (PPARγ), Fatty acid binding protein 4 (FABP4), CCAAT/enhancer binding protein alpha (C/EBPα) and Perilipin-1 (PLIN1) Fatty acid synthase (FASN) and Acetyl-CoA carboxylase (ACC). Data are presented as means ± SEM and analyzed by one-way ANOVA (**B**–**D** and **G,** *n* = 3–4 per group) or two-way ANOVA (**E**, *n* = 4 per group) followed by post-hoc testing. Scale bars: 200 μm

It has been reported that the regulation of osteogenesis and adipogenesis from BMSCs is reciprocal [18]. Since loss of *HSPB7* inhibited osteogenic differentiation, we explored whether *HSPB7* silencing could promote adipogenesis. As shown by Oil Red O staining, knockdown of *HSPB7* facilitated adipogenic differentiation (Fig. 4A, B) without influencing adipocyte proliferation, which was determined by assessing cell numbers (Fig. 4C) and calculating absorbance of individual cells (Fig. 4D). Consistently, elevated levels of adipocyte marker genes including *PPARG*, *FABP4*, *LPL* and *PLIN1* were observed following *HSPB7* silencing (Fig. 4E), and the expression levels of key transcription factors including Peroxisome proliferator-activated receptor gamma (PPARγ) and CCAAT/enhancer binding protein alpha (C/EBPα), as well as Perilipin-1 and Fatty acid binding protein 4 (FABP4) were further confirmed at protein level (Fig. 4F–G). Additionally, the expression of lipogenic proteins such as Fatty acid synthase (FASN) and Acetyl-CoA carboxylase (ACC) were increased up to fivefold following *HSPB7* knockdown (Fig. 4F-G). Collectively, our findings indicate that reduced levels of *HSPB7* lead to enhanced adipogenesis.

### HSPB7 overexpression in BMSCs enhances osteogenic differentiation and mineralization, but did not affect adipogenesis

**Fig. 5:**
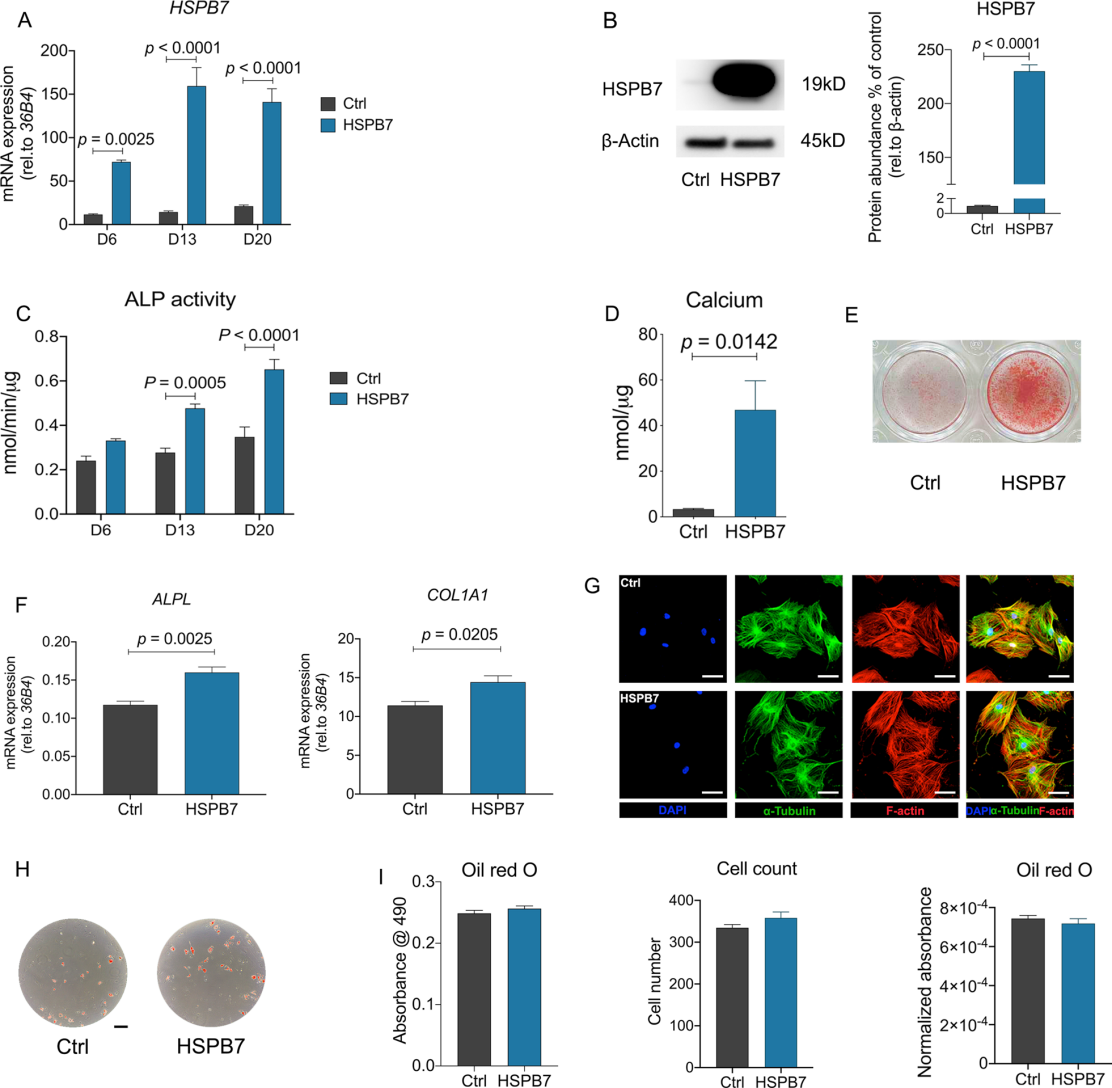
*HSPB7* overexpression in BMSCs enhances osteogenic differentiation and mineralization, but not adipogenesis. **A** *HSPB7* overexpression was assessed by quantification of HSPB7 mRNA expression at different time points. **B** Representative images and quantitative expression of HSPB7 were assessed by Western blot using HSPB7 antibody on day 3 following osteogenic induction. Full-length blots are presented in Additional file 7: Fig. S6. **C** ALP activity was evaluated at multiple time points with osteogenic media. **D**, **E** Osteoblast mineralization was assessed by calcium deposition assay (**D**) and Alizarin Red S staining (**E**) after 3 weeks osteogenic induction. **F** Osteogenesis-related gene were evaluated at different time points with osteogenic induction. **G** Representative images of immunostainings against F-actin (phalloidin-rhodamine), α-tubulin (Alexa Fluor 488) and Nuclei (DAPI) on day 14 following osteogenic induction. **H**, **I** Representative images (**H**) and quantitative data (**I**) of Oil Red O staining performed after 14 days with adipogenic induction. Total cell number was determined by DAPI staining. Quantitative data of Oil Red O staining was adjusted by cell number. Data are presented as means ± SEM and analyzed by two-tailed Student’s *t*-test ANOVA (**B**, **D**, **F** and **I**, *n* = 3–4 per group) or two-way ANOVA (**A** and** C**, *n* = 4 per group) followed by post-hoc testing. Scale bars: 200 μm

To further provide insights into the role of *HSPB7* during osteogenic differentiation, we examined the effect of *HSPB7* gain-of-function on osteogenic differentiation and mineralization in BMSCs. qRT-PCR showed that *HSPB7* expression is significantly increased at all investigated time points (Fig. 5A), and this enhanced expression was confirmed by Western blot (Fig. 5B). In contrast to *HSPB7* knockdown, cell viability was not affected by *HSPB7* overexpression (Additional file 2: Fig. S1B). Following osteogenic induction, ALP activity was increased when *HSPB7* was overexpressed (Fig. 5C). Similarly, calcium deposition was also elevated following *HSPB7* overexpression, as revealed by calcium quantitation and Alizarin Red S staining at day 21 (Fig. 5D–E). Moreover, overexpression of *HSPB7* in BMSCs facilitated the expression of osteogenic marker genes, including ALPL and COL1A1 (Fig. 5F). However, cell morphology and cytoskeleton rearrangement looked similar between control and *HSPB7* overexpression groups (Fig. 5G).

Considering the reciprocal role of osteogenesis and adipogenesis, and the observation that gain-of-function of *HSPB7* favors osteogenic differentiation and mineralization, we wondered whether the adipogenic differentiation of BMSCs could be affected by *HSPB7* overexpression. In contrast to the osteogenic lineage effects, overexpression of *HSPB7* in BMSCs failed to alter the lineage commitment toward adipocytes, as evidenced by unaffected Oil Red O staining (Fig. 5H, I). Collectively, these findings suggest that *HSPB7* overexpression has a positive effect on osteogenic differentiation and mineralization, but does not influence adipogenesis in BMSCs.

### The ability of HSPB7 to enhance osteogenic differentiation depends on the N- and C-terminal domains

**Fig. 6:**
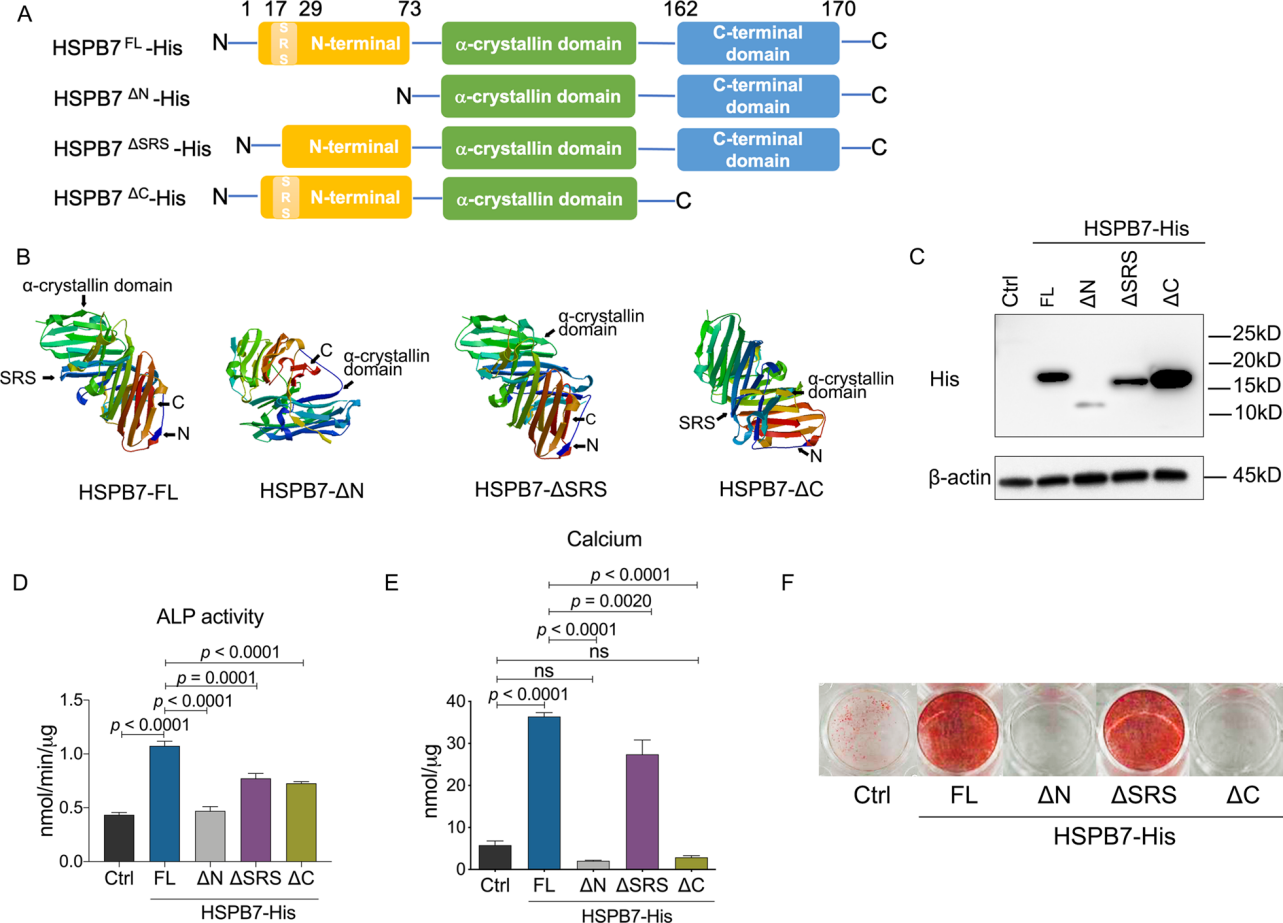
The ability of HSPB7 to enhance osteogenic differentiation depends on *N*- and *C*-terminal domains. **A** A schematic representation of full-length HSPB7 and its truncated mutants. His tag is located at the *C* terminal in each construct **B** 3D structures of FL HSPB7 and its truncated mutants generated by SWISS-MODEL (https://swissmodel.expasy.org). These models were predicated with reference to the template of 4jut.1.A and were selected on the basis of GMQE and QMEANDisCo global scores. **C** BMSCs expressing the indicated HSPB7 constructs were immunoblotted with anti-histidine antibody in non-differentiating conditions. Control samples were transduced with dsRED. Full-length blots are presented in Additional file 7: Fig. S6. **D** ALP activity was evaluated in BMSCs expressing dedicated constructs under osteogenic induction at day 13. **E**, **F** Osteoblast mineralization was assessed by calcium deposition assay (**E**) and Alizarin Red S staining (**E**) after three weeks osteogenic induction in BMSCs. Abbreviations: Δ*N*, *N*-terminus deletion; Δ*C*, *C*-terminus deletion; SRS, serine-rich sequence stretch; FL, full-length. Data are presented as means ± SEM and analyzed by one-way ANOVA (*n* = 4 per group) followed by post-hoc testing

sHSPs are characterized by a conserved α-crystallin domain flanked by a flexible sized *N*- and *C*-terminus that is mostly divergent among family members. However, the role of these functional domains in controlling the differentiation behavior of BMSC is unknown. Therefore, we explored the role of structural characteristics in *HSPB7* toward osteogenic differentiation and mineralization. Apart from deleting the *N*- or *C*-terminal domains (Δ*N* or Δ*C*, respectively), we also focused our attention on the serine-rich stretch sequence (SRS, Δ17–29) within the *N*-terminal domain, which has been considered as a critical segment for proper *HSPB7* function [30] (Fig. 6A). Based on sequence analyses, structure prediction showed that truncated proteins have different organization and 3D configuration compared to the original *HSPB7* protein (Fig. 6B). To further explore the function of each domain, we then overexpressed them by lentiviral transduction and validated their expression and size by immunoblotting (Fig. 6C). The intensity of the bands may reflect protein instability or degradation as gene expression of these overexpression constructs was more or less similar (Additional file 4: Fig. S3). Compared to full-length overexpression, deletion of the SRS and *N*-terminus resulted in a decrease in ALP activity (Fig. 6D). *C*-terminus deletion also led to a decrease in ALP activity despite the strongly increased protein level compared to full-length *HSPB7* (Fig. 6C–D). Interestingly, deletion of SRS led to a marginal decrease in mineralization while an absolute lack of mineralization was observed when the *N*-terminus was completely deleted (Fig. 6E, F). Also, deletion of the *C*-terminus domain led to a complete block of mineralization, i.e., a mineralization below control level (Fig. 6E, F). To determine whether impaired mineralization was caused by altered subcellular localization of deletion mutants, we performed immunostainings, using His antibody to detect full-length and truncated *HSPB7* proteins. As shown in Additional file 5: Fig. S4, all three deletion mutants had similar subcellular localization as full-length *HSPB7*. Collectively, these data indicate *N* and *C*-terminus are dominant domains, while SRS is not involved in *HSPB7*-regulated osteogenesis.

### Activin A blockage overcomes the HSPB7 knockdown-mediated osteogenic inhibition

**Fig. 7:**
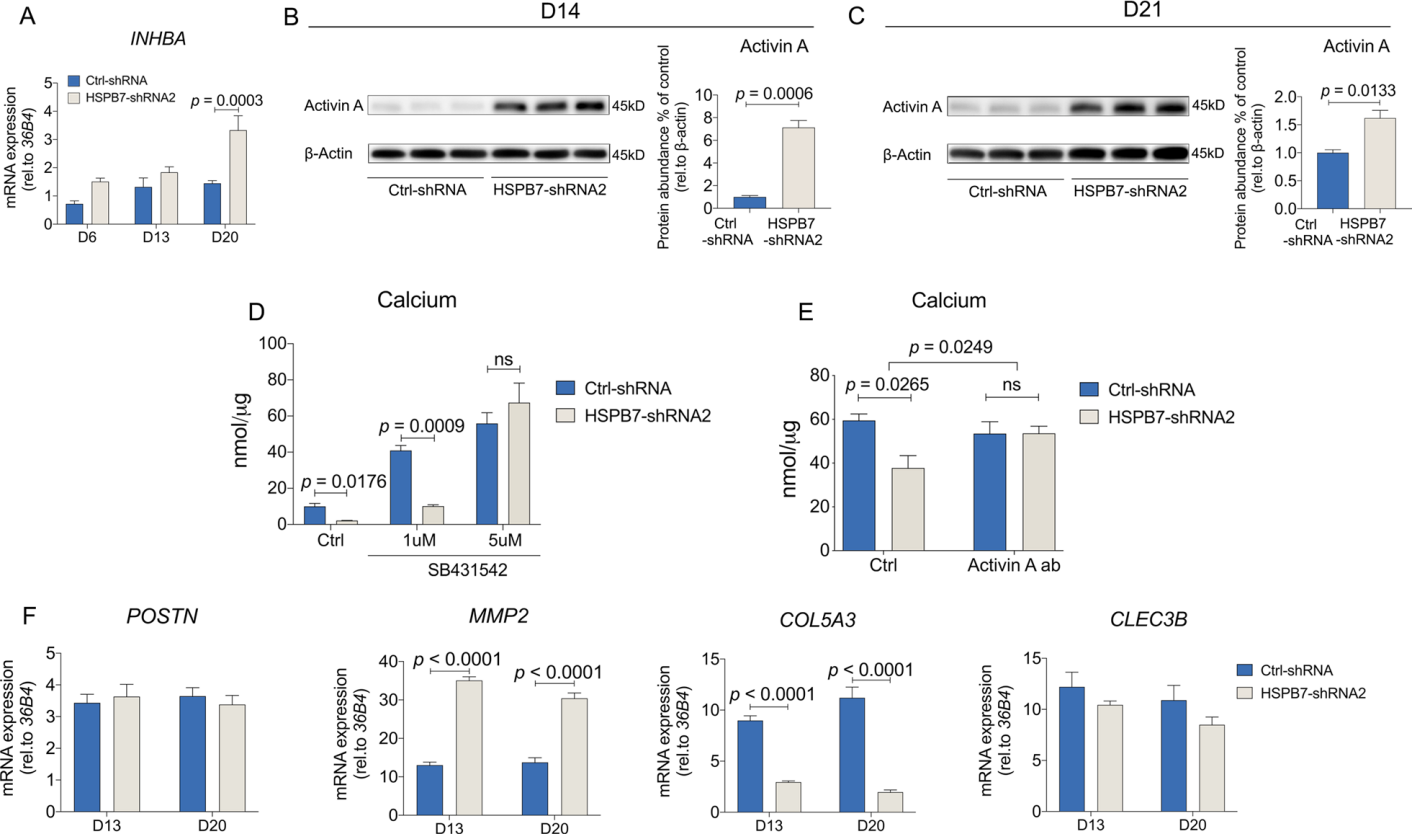
Blocking Activin A overcomes *HSPB7* silencing-mediated osteogenic inhibition. **A** *INHBA* mRNA expression was assessed by qRT-PCR following *HSPB7* silencing at different time points following osteogenic induction. **B**, **C** *INHBA* mRNA expression was assessed by Western blot following *HSPB7* silencing at day 14 (**B**) and day 21 (**C**) following osteogenic induction. Full-length blots are presented in Additional file 7: Fig. S6. **D** Osteoblast mineralization was assessed by calcium deposition assay after three weeks osteogenic induction. BMSCs were induced to osteogenic differentiation following *HSPB7* silencing in the presence of SB431542 (**D**) or neutralizing Activin A antibodies (**E**). **F** ECM genes were evaluated by qRT-PCR following *HSPB7* silencing at day 13 and day 20. Data are presented as means ± SEM and analyzed by two-tailed Student’s *t*-test (**B**–**C,** *n* = 3 per group) or two-way ANOVA (**A**, **D**–**F**, *n* = 4 per group) followed by post-hoc testing

Earlier work by others had proposed an interaction between *HSPB7* and Activin A [31]. Besides, our previous findings have shown an inhibitory effect of Activin A on osteogenesis [32]. Therefore, we postulated that Activin A interacts with *HSPB7* during osteogenic differentiation. ShRNA2 was selected to investigate the downstream effect as it showed the strongest knockdown of *HSPB7* (Fig. 2A, B). The increased expression of Activin A was observed throughout the entire osteogenic differentiation process and knockdown of *HSPB7* significantly stimulated Activin A expression encoded by *INHBA* (Fig. 7A). Such effects were more pronounced when evaluating Activin A expression using immunoblotting at late stages of osteoblast differentiation (Fig. 7B, C). On the other hand, Activin A expression showed a biphasic pattern following *HSPB7* overexpression with an early increase on day 6 followed by a decline from day 13 onward, and at day 20, a significant down-regulation of *INHBA* was observed when *HSPB7* was continually overexpressed (Additional file 6: Fig. S5A), consistent with the findings in Immunoblots (Additional file 6: Fig. S5B, C).

We next examined whether Activin A is a downstream target involving *HSPB7*-mediated osteogenic effect. Activin A signaling antagonist SB431542 prevented the *HSPB7* knockdown-mediated inhibition of mineralization (Fig. 7D). To further confirm this finding, we used Activin A antibodies to neutralize secreted Activin A. As shown in Fig. 7E, *HSPB7* silencing-mediated inhibition of osteoblast mineralization was abolished by Activin A antibody treatment (Fig. 7E). Collectively, these data demonstrate that Activin A plays a critical role in HSPB7-mediated osteogenesis.

Our previous work had suggested that Activin A negatively regulates osteoblast mineralization through altering the maturation of the extracellular matrix (ECM) [33]. Based on that, we selected several ECM genes and assessed whether the expression was changed during later stages of osteoblast differentiation (Fig. 7F and Additional file 6: Fig. S5D). *COL5A3* and *MMP2* were strongly regulated by *HSPB7* silencing (Fig. 7F), while these effects were not observed in *HSPB7* overexpressing cells (Additional file 6: Fig. S5D). One the other hand, *POSTN* and *CLEC3B* were significantly changed during late (day 20) osteoblast differentiation following *HSPB7* overexpression (Additional file 6: Fig. S5D), but did not reveal differences following *HSPB7* knockdown (Fig. 7F).

## Discussion

**Fig. 8:**
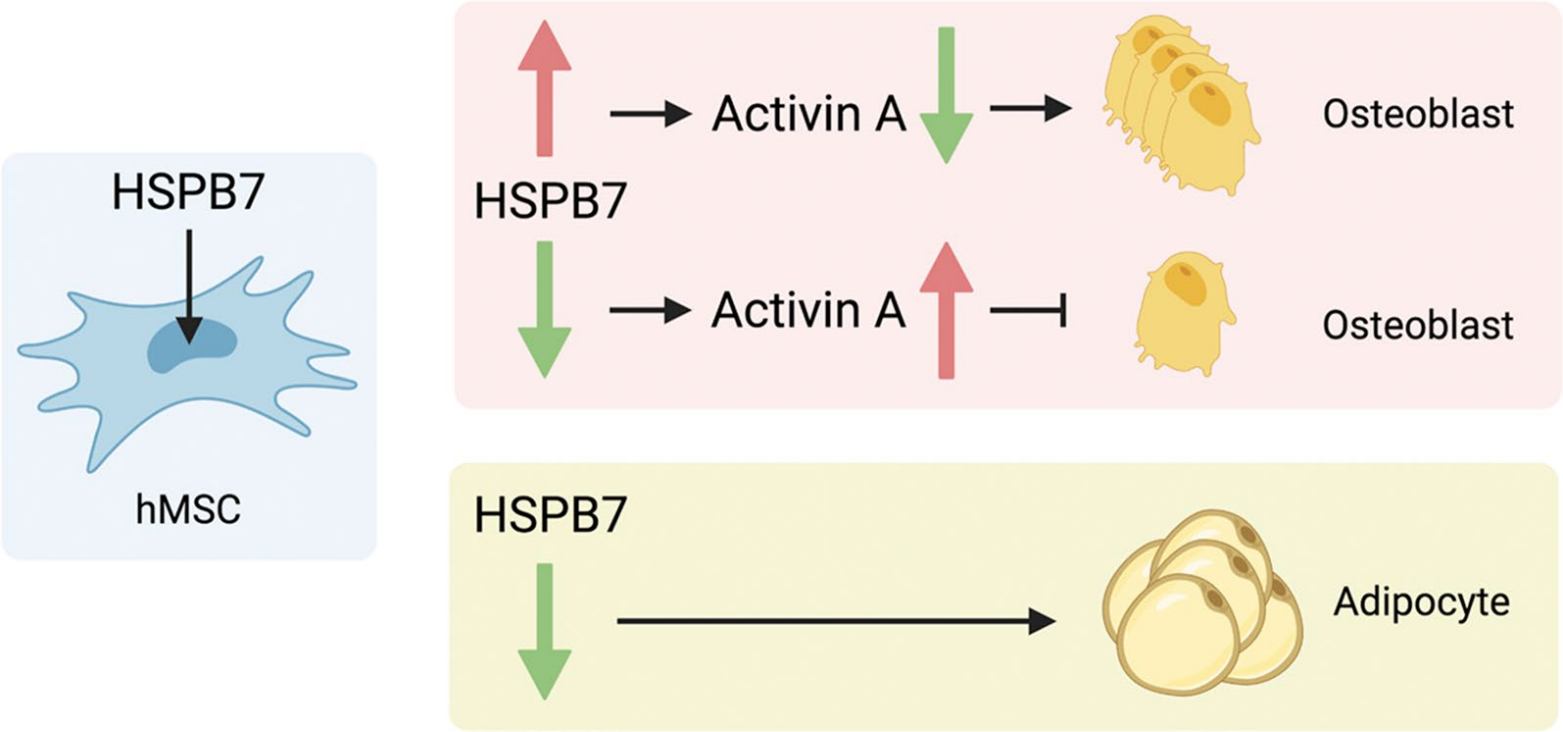
A schematic diagram of HSPB7-regulated osteogenesis and adipogenesis. HSPB7 regulates the osteogenic differentiation through Activin A

In the present study, we demonstrate that *HSPB7* is a molecular switch in regulating osteogenic and adipogenic differentiation of BMSCs (Fig. 8). Our gain- and loss-of-function studies showed that HSPB7 intrinsically promotes osteogenesis. Moreover, knockdown of *HSPB7* stimulated adipogenesis, further emphasizing the importance of HSPB7 for BMSC differentiation. Meanwhile, we show that HSPB7 expression is indispensable for osteogenic induction by affecting cell morphology and cytoskeletal reorganization. Complete deletion of the *N*- or *C*-terminus of HSPB7 leads to absence of mineralization indicating that they are essential domains for osteogenesis. Our data indicate Activin A as a downstream target in the HSPB7 cascade controlling osteogenic differentiation. These findings pave the way for the identification of molecular switches, which mediate the inverse relationship between osteogenesis and adipogenesis when designing therapeutic strategies toward lineage commitment-related diseases.

HSPB7 was discovered as a cardiovascular-related chaperone, but recent studies revealed that HSPB7 participates in several physiological and pathological processes as it is ubiquitously expressed in other tissues than originally anticipated [30]. In addition to the high expression profile in heart and muscle, HSPB7 is strongly upregulated during osteogenic differentiation especially in late stages compared with undifferentiation stage (day 0). Structurally, HSPB7 forms hetero-oligomeric complexes with HSPB8 through interaction of their C-termini [34]. Downregulation of *HSPB8* led to reduction of ALP activity and mineral deposition, as well as the expression of osteogenic markers in dental pulp stem cell [22]. Therefore, it is plausible to speculate that similar functional characteristics and activities could be shared between HSPB7 and HSPB8 in MSCs. Our observations following HSPB7 knockdown and osteogenic differentiation are in line with this notion and demonstrate an important function for HSPB7 in osteogenesis. In addition, the positive role of HSPB7 in osteogenesis and mineralization is further supported by our overexpression studies.

It has been demonstrated that adipocyte induction factors suppress osteogenesis, and conversely, osteoblast induction factors hamper adipogenesis [18]. In fact, various external cues including physical, chemical and biological signals could influence the balance between adipogenic and osteogenic differentiation. In this study, we showed that knockdown of HSPB7 increases adipogenic competency by promoting PPARγ and C/EBPα, which are master regulators of the adipocyte phenotype. Consistent with our findings, the expression of PPARγ and C/EBPα are maintained at high levels throughout the entire differentiation process and cooperate to regulate a number of adipogenic proteins such as FABP4 and Perilipin-1 [18, 35]. In addition, PPARγ is involved in de novo lipogenesis by mediating Acetyl-CoA carboxylase [36], which acts as a rate-limiting enzyme and catalyzes the production of malonyl-CoA used as an essential substrate for Fatty acid synthase. The disruption of HSPB7 has no direct impact on cell number in the adipogenic condition, highlighting that HSPB7 acts directly on differentiation and lipid synthesis in the adipocytes. Of note, as opposed to *HSPB7* overexpression not affecting cell proliferation, *HSPB7* silencing slowed down cell proliferation, suggesting that different mechanisms are involved in HSPB7-mediated osteogenesis. In contrast to our observations that knockdown of HSPB7 enhances adipogenic differentiation and impairs osteogenic differentiation, Jin et al. [20] observed the opposite trend in adipogenesis and osteogenesis using human adipose tissue-derived stem cells. The discrepant observation with our work may, apart from differences in methodologies, be due to different molecular mechanisms underlying the lineage commitment process between BMSCs versus adipose tissue-derived MSCs (ASC), as ASCs have significantly higher adipogenic capacity, while BMSCs have pronounced osteogenic capacity shown in a donor-matched study [37]. Collectively, our data demonstrate a role for HSPB7 in balancing the osteoblast and adipocyte differentiation potential from bone marrow MSCs.

A number of studies have demonstrated that actin filament-mediated cell shape changes are required for MSC differentiation [38, 39]. Moreover, actin has roles in determining nuclear shape, cell spreading, and cell stiffness, which eventually influence MSC fate decision [15]. In this study, we observed aberrant actin filament networks along with disrupted osteogenic differentiation and mineralization induced by *HSPB7* silencing. Remarkably, rather than the classic cuboidal-shaped osteoblasts with actin stress fibers, BMSCs developed a spindle shape-like fibroblast morphology with thin microfilaments across the cytoplasm after 14 days of osteogenic induction in the absence of HSPB7. Consistent with our findings, genetic studies have reported that cardiacspecific deficiency of *Hspb7* in mice show longer actin/thin filaments with abnormal actin filament bundles in sarcomeres, which is a contractile unit of striated muscle [40]. In addition, loss of HSPB7 in mouse cardiomyocytes upregulates the expression of Connexin43, which plays a critical role in osteogenesis and cell–cell communication in the skeleton [41, 42].

It has been reported that HSPB7 is the most potent molecular chaperone among the sHSP family in suppressing the aggregation of polyglutamine-containing proteins, which cause neurodegenerative conditions including Huntington’s disease and Kennedy’s disease [30], and most importantly, this anti-aggregation function is conserved among species [43]. The main structural hallmarks that make HSPB7 unique within the sHSP family are the SRS located near the *N*-terminus (13 amino acids) and the conserved *C*-terminal region composed of 9 residues [30]. The complete deletion of the *N*-terminal domain leads to the abrogation of its activity, suggesting the indispensable role for this protein domain [30]. Based on these previous findings, we generated different deletion constructs and investigated which parts of HSPB7 were required for osteoblastic differentiation and mineralization. Although the mechanism in promoting osteogenesis is probably different from neurodegenerative diseases, the deletion of the *N*-terminus renders the protein less functional, abrogating osteogenic stimulation induced by full-length HSPB7. In contrast to the negligible role of the *C*-terminal deletion in Huntington’s disease [30], our data shows that the osteogenic effect as indicated by ALP activity and calcium deposition, were less strong following deletion of the SRS in comparison with full-length HSPB7, despite increased amounts of protein. These results further underscore the structural importance of these specific domains within HSPB7.

Mechanistically, we found that Activin A is a downstream target of HSPB7-mediated osteogenic effects as knockdown of *HSPB7* resulted in upregulation of Activin A expression. On the basis of these data, it can be hypothesized that HSPB7 stimulates the osteogenic capacity through mediating Activin A expression. This hypothesis is substantiated by the changes in expression levels of ECM genes. The altered ECM composition could impair the production of matrix vesicles, and further osteoblast mineralization [32]. In addition, blocking Activin A activity either by SB431542 [44], a selective inhibitor of Activin A signaling or by a neutralizing antibody treatment, rescued osteoblast mineralization suppressed by *HSPB7* silencing. These findings are in accordance with previous reports that Activin A treatment suppresses osteogenesis leading to changes in ECM gene expression and significant reduction of the mineralization capacity [32, 45]. However, we did not observe difference in expression levels of Activin A following *HSPB7* overexpression. Future studies are needed to investigate the molecular machinery between HSBP7 and Activin A, and to determine the extent to which Activin A is involved in HSPB7-enhanced osteogenesis.

## Conclusions

Osteoporosis is one of the most common bone metabolic diseases partially caused by aberrant lineage specification of BMSCs [46]. This abnormal lineage commitment is characterized by diminished osteoblast formation accompanied by excessive adipocyte accumulation in the bone marrow cavity [47, 48]. In this study, we revealed that HSPB7 plays a positive role in driving osteoblastic differentiation, and with the capability in maintaining the osteo-adipogenesis balance involving cytoskeletal changes and interplay with Activin A and modulation of ECM, it holds great promise as a potential therapeutic target in bone metabolic diseases and further supports a role for the sHSPs family in stem cell control and differentiation.

## Supplementary Information

**Additional file 1**: **Table S1**. List of shRNAs used for HSPB7. **Table S2**. Primer sequences used to generate deletion constructs. **Table S3**. Primer sequences used for q-RT PCR in this study

**Additional file 2**: **Fig. S1**. HSPB7 silencing in BMSCs affects cell viability. Cell viability was evaluated following HSPB7 knockdown or HSPB7 overexpression in the presence of osteogenic induction at indicated time points using CCK-8 assay. Data are presented as means ± SEM and analyzed by two-way ANOVA followed by post-hoc testing.

**Additional file 3**: **Fig. S2**. HSPB7 silencing in BMSCs affects cytoskeleton reorganization. A–C Representative images of immunostaining for F-actin, α-tubulin and nuclei at day 3, day 7 and day 20 following osteogenic induction. Arrows indicate spindle-shaped fibroblast-like cells. Scale bars: 200 μm.

**Additional file 4**: **Fig. S3**. HSPB7 gene expression of lentivirally transduced deletion constructs. A–B HSPB7 mRNA expression was assessed by qRT-PCR following transduction with deletion constructs. His primers were used to amplify exogenous expression of HSPB7, while primers targeting coding DNA sequence were used to amplify both endogenous and exogenous expression of HSPB7. Data are presented as means ± SEM and analyzed by one-way ANOVA followed by post-hoc testing.

**Additional file 5**: **Fig. S4**. Overexpression of HSPB7 deletion mutants have similar intercellular localization as full-length HSPB7. BMSCs expressing the indicated deletion constructs were immunostained with His and F-actin, and nuclei after 3 days osteogenic induction. Scale bars: 200 μm.

**Additional file 6**: **Fig. S5**. Overexpression of HSPB7 affects the expression of extracellular matrix genes. A INHBA mRNA expression was assessed by qRT-PCR following HSPB7 overexpression at multiple time points. B–C Representative images and quantitative expression of activin A were assessed by Western blot at day 14 and day 21 following osteogenic induction. Full-length blots are presented in Additional file 7: Fig. S6. D ECM genes were evaluated by qRT-PCR following HSPB7 overexpression at day 13 and day 20. Data are presented as means ± SEM and analyzed by one-way ANOVA followed by post-hoc testing or two-tailed Student’s t-test.

**Additional file 7**: **Fig. S6** Full-length blots.

## Data Availability

The datasets generated and analyzed during the current study are available from the corresponding author on reasonable request.

## Abbreviations

BMSC: Bone marrow-derived mesenchymal stromal cells
sHSPs: Small heat shock proteins
VEGF: Vascular endothelial growth factors
TGF: Transforming growth factor
FGF: Fibroblast growth factor
α-MEM: Alpha minimum essential medium
PBS: Phosphate-buffered saline
shRNA: Short hairpin RNA
RT: Room temperature
ALP: Alkaline phosphatase
*p*NPP: *p*-Nitrophenyl phosphate
*p*NP: *p-*Nitrophenol
PPARγ: Proliferator-activated receptor gamma
C/EBPα: CCAAT/enhancer binding protein alpha
FABP4: Fatty acid binding protein 4
FASN: Fatty acid synthase
ACC: Acetyl-CoA carboxylase
Δ*N*: *N*-Terminal domain deletion
Δ*C*: *C*-Terminal domain deletion
ΔSRS: Serine-rich stretch sequence deletion
ECM: Extracellular matrix
ASC: Adipose tissue-derived mesenchymal stromal cells
