# Supplementary material for: HSPB7 oppositely regulates human mesenchymal stromal cell-derived osteogenesis and adipogenesis
Source: Stem Cell Res Ther. 2023 May 11;14:126. doi: 10.1186/s13287-023-03361-0 (PMC10173662; doi:10.1186/s13287-023-03361-0)
Supplement: Supplementary file 1 — Additional file 1: Table S1. List of shRNAs used for HSPB7. Table S2. Primer sequences used to generate deletion constructs. Table S3. Primer sequences used for q-RT PCR in this study [file 13287_2023_3361_MOESM1_ESM.docx]

Table S1. List of shRNAs used for HSPB7

| Target gene | ID | Target sequence |
| --- | --- | --- |
| HSPB7 | shRNA1 | GCAGGCAACATCAAGACCCTA |
|  | shRNA2 | CATGAACACCTTCGCTCACAA |
| Nontargeting shRNA | ID | Oligonucleotide sequence |
| Ctrl | SHC002 | CCGGCAACAAGATGAAGAGCACCAACTCGAGTTGGTGCTCTTCATCTTGTTGTTTTT |

Table S2. Primer sequences used to generate deletion constructs

| Name | Sequence |
| --- | --- |
| HSPB7 Δ2-73aa-forward | AACATCAAGACCCTAGGAGACGC |
| HSPB7 Δ2-73aa-reverse | CATGGTGAAGGGGGCGGC |
| HSPB7 Δ17-29aa-forward | GCCTCCCGTGCCCTCCCG |
| HSPB7 Δ17-29aa-reverse | ATGGAAACTTCTCTCCGCTCGGAAGGTG |
| HSPB7 Δ162-170aa-forward | CATCACCATCACCATCAC |
| HSPB7 Δ162-170aa-reverse | CTGGACGTGTTCTGTATG |

Table S3. Primer sequences used for q-RT PCR in this study

| Name | Sequence |
| --- | --- |
| HSPB7-CDS-forward | TGCGGGCTGAGAAGCTGG |
| HSPB7-CDS-reverse | CGGATAGTGAGGCTGCCG |
| PPARG- forward | TCTCAAACGAGAGTCAGCCT |
| PPARG- reverse | CACGGAGCTGATCCCAAAGT |
| FABP4- forward | TACTGGGCCAGGAATTTGAC |
| FABP4- reverse | GGACACCCCCATCTAAGGTT |
| PLIN1- forward | CCTGCCTTACATGGCTTGTT |
| PLIN1- reverse | ATTCTCCTGCTCAGGGAGGT |
| LPL-forward | TTGGAGAAGCTATCCGCGTG |
| LPL-reverse | CGTGGGAGCACTTCACTAGC |
| HSPB7-His- forward | AGGACGGCAGCCTCACTATC |
| HSPB7-His- reverse | TCATTACTAGTGATGGTGATGGTGATG |
