# Supplementary figures and images for: HSPB7 oppositely regulates human mesenchymal stromal cell-derived osteogenesis and adipogenesis
Source: Stem Cell Res Ther. 2023 May 11;14:126. doi: 10.1186/s13287-023-03361-0 (PMC10173662; doi:10.1186/s13287-023-03361-0)

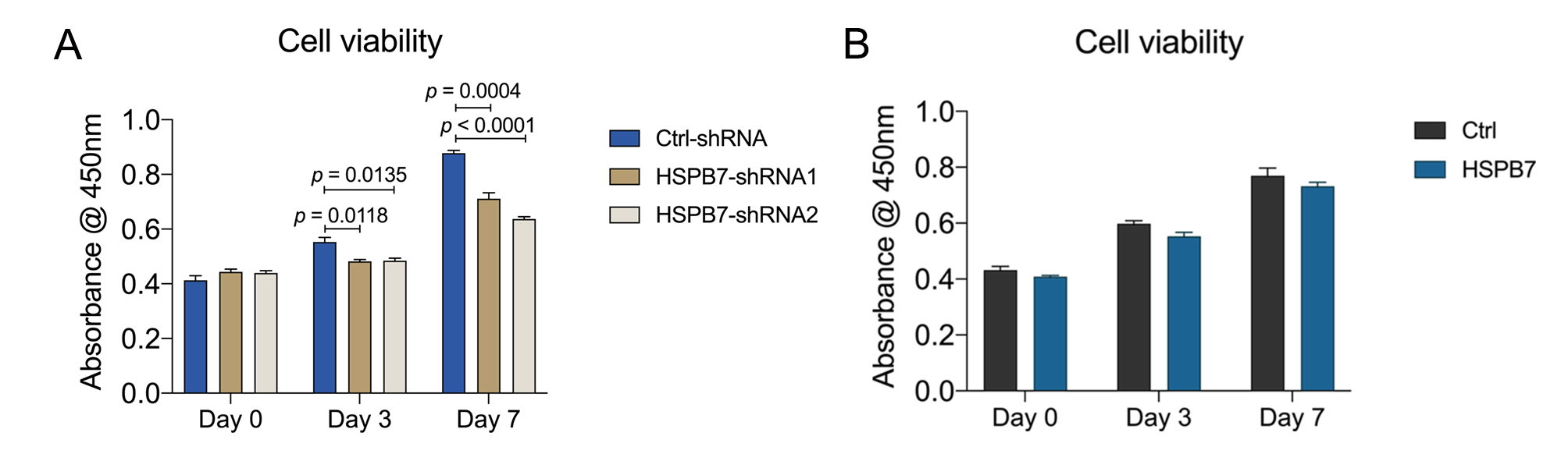

Supplement: Supplementary file 2 — Additional file 2: Fig. S1. HSPB7 silencing in BMSCs affects cell viability. Cell viability was evaluated following HSPB7 knockdown or HSPB7 overexpression in the presence of osteogenic induction at indicated time points using CCK-8 assay. Data are presented as means ± SEM and analyzed by two-way ANOVA followed by post-hoc testing. [file 13287_2023_3361_MOESM2_ESM.tif]

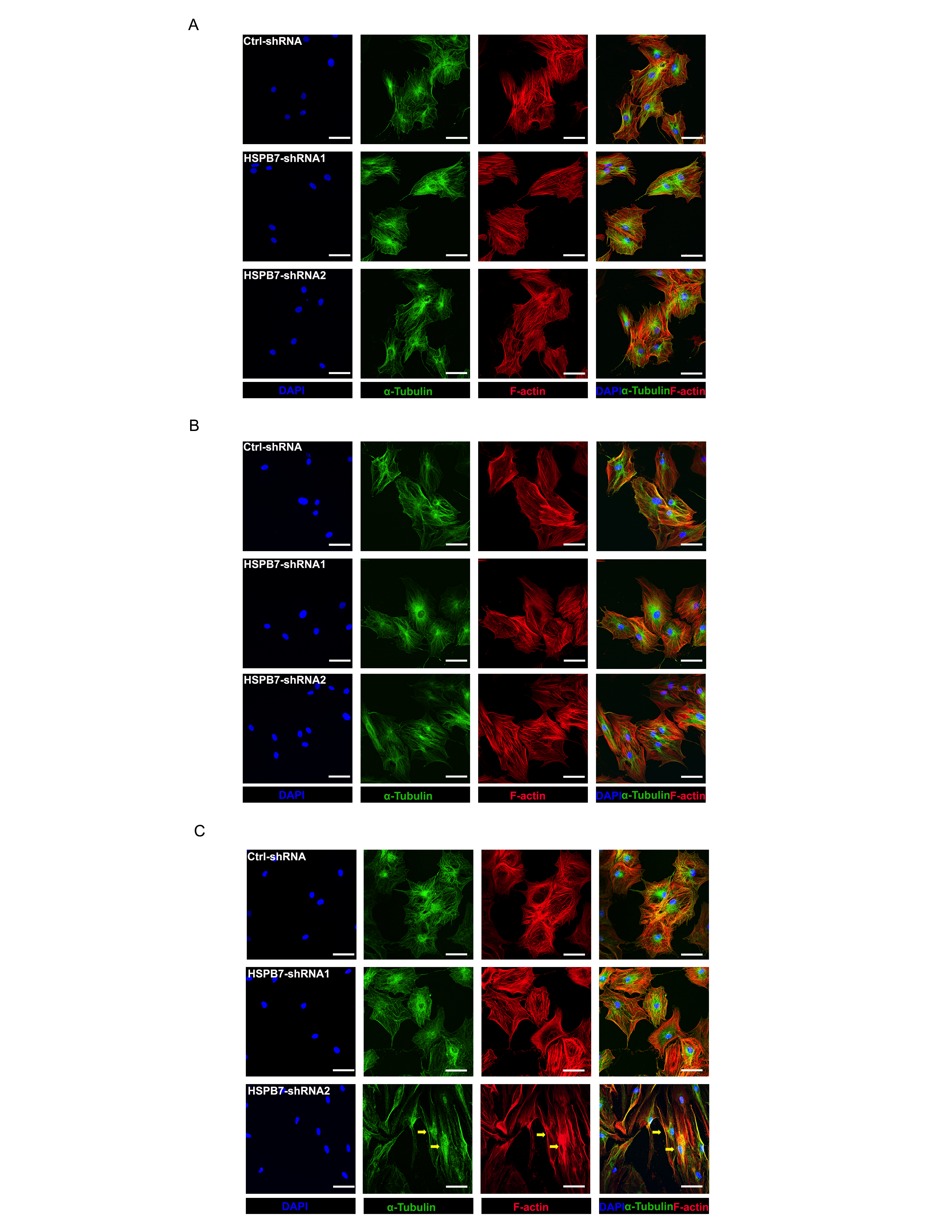

Supplement: Supplementary file 3 — Additional file 3: Fig. S2. HSPB7 silencing in BMSCs affects cytoskeleton reorganization. A–C Representative images of immunostaining for F-actin, α-tubulin and nuclei at day 3, day 7 and day 20 following osteogenic induction. Arrows indicate spindle-shaped fibroblast-like cells. Scale bars: 200 μm. [file 13287_2023_3361_MOESM3_ESM.tif]

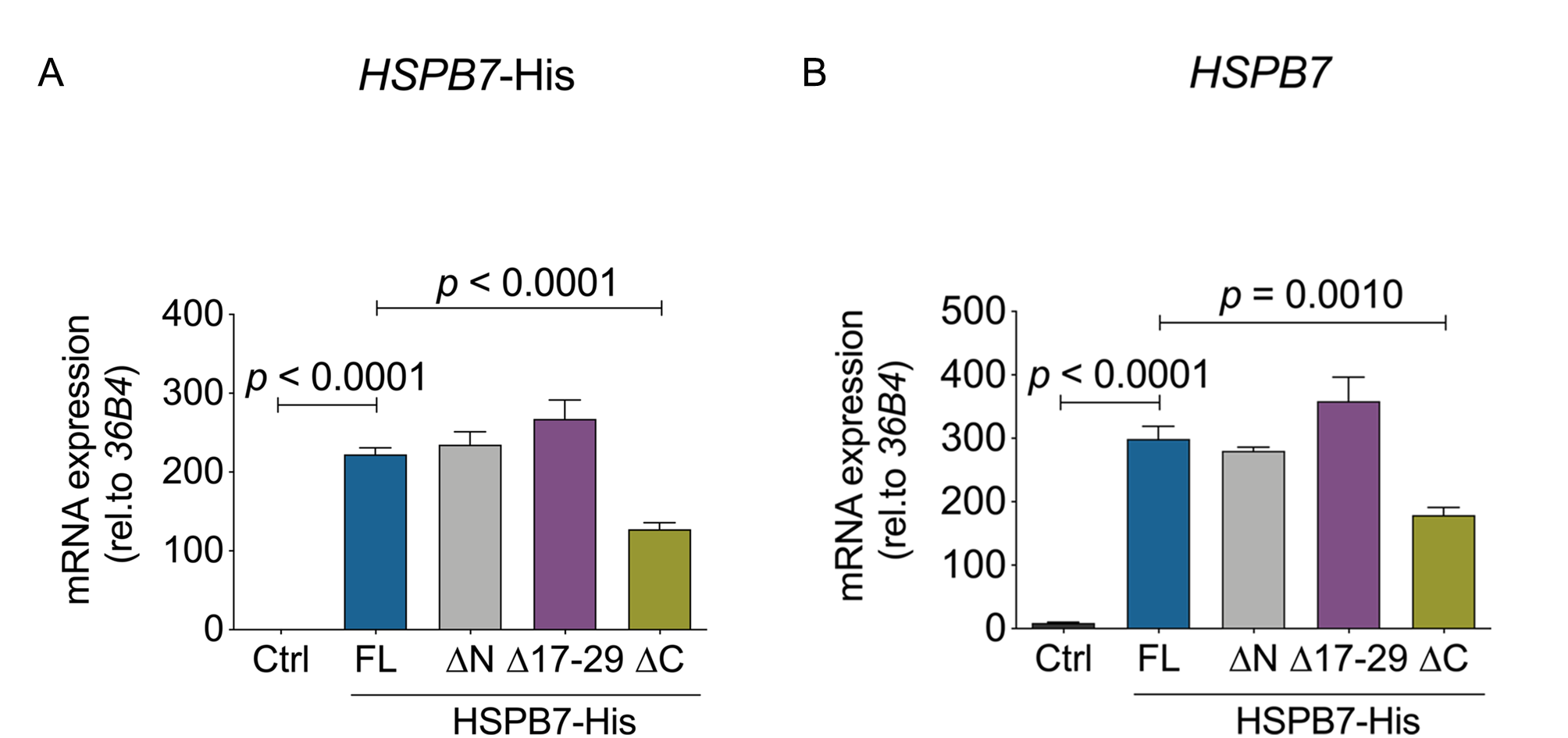

Supplement: Supplementary file 4 — Additional file 4: Fig. S3. HSPB7 gene expression of lentivirally transduced deletion constructs. A–B HSPB7 mRNA expression was assessed by qRT-PCR following transduction with deletion constructs. His primers were used to amplify exogenous expression of HSPB7, while primers targeting coding DNA sequence were used to amplify both endogenous and exogenous expression of HSPB7. Data are presented as means ± SEM and analyzed by one-way ANOVA followed by post-hoc testing. [file 13287_2023_3361_MOESM4_ESM.tif]

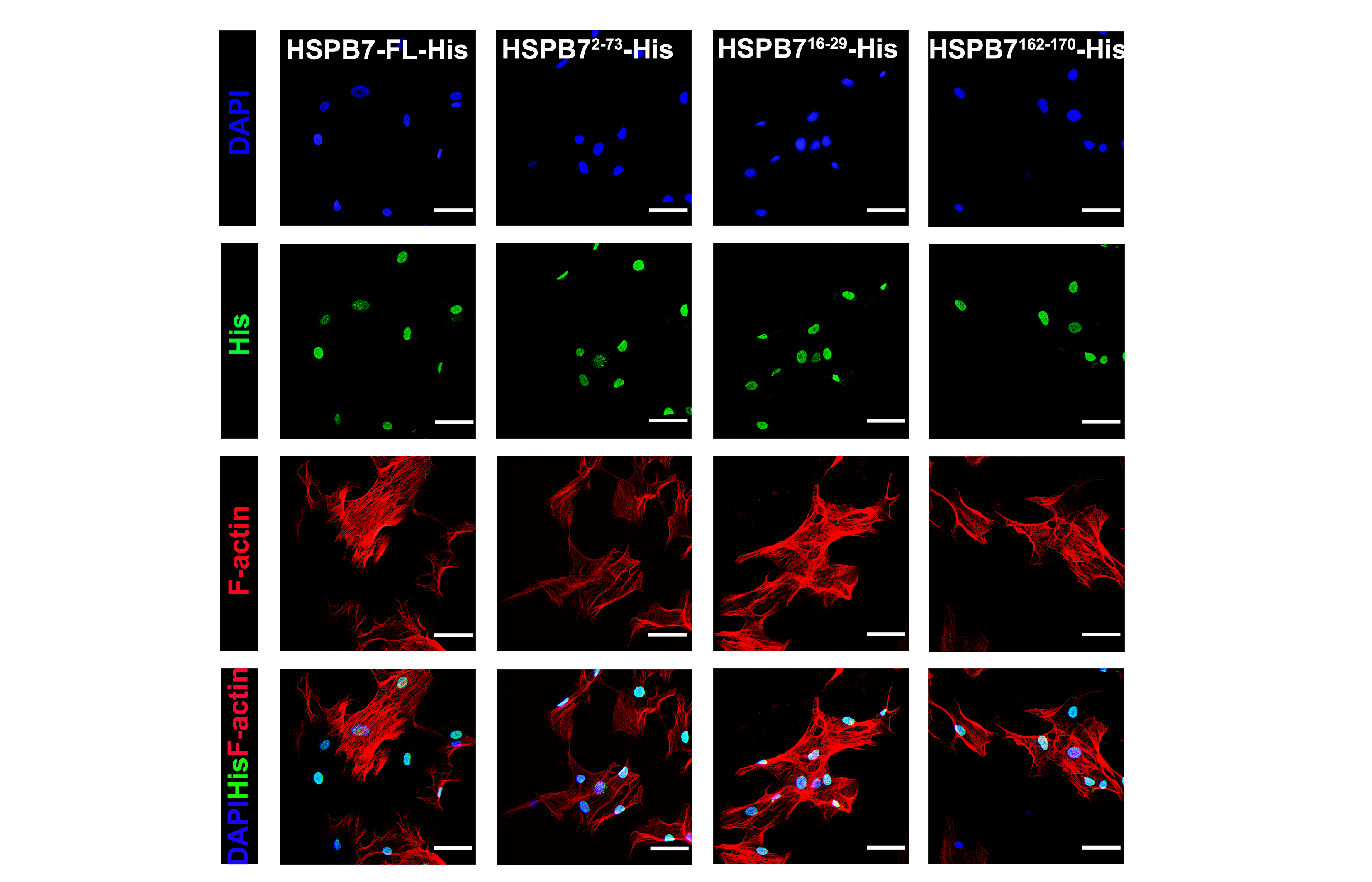

Supplement: Supplementary file 5 — Additional file 5: Fig. S4. Overexpression of HSPB7 deletion mutants have similar intercellular localization as full-length HSPB7. BMSCs expressing the indicated deletion constructs were immunostained with His and F-actin, and nuclei after 3 days osteogenic induction. Scale bars: 200 μm. [file 13287_2023_3361_MOESM5_ESM.tif]

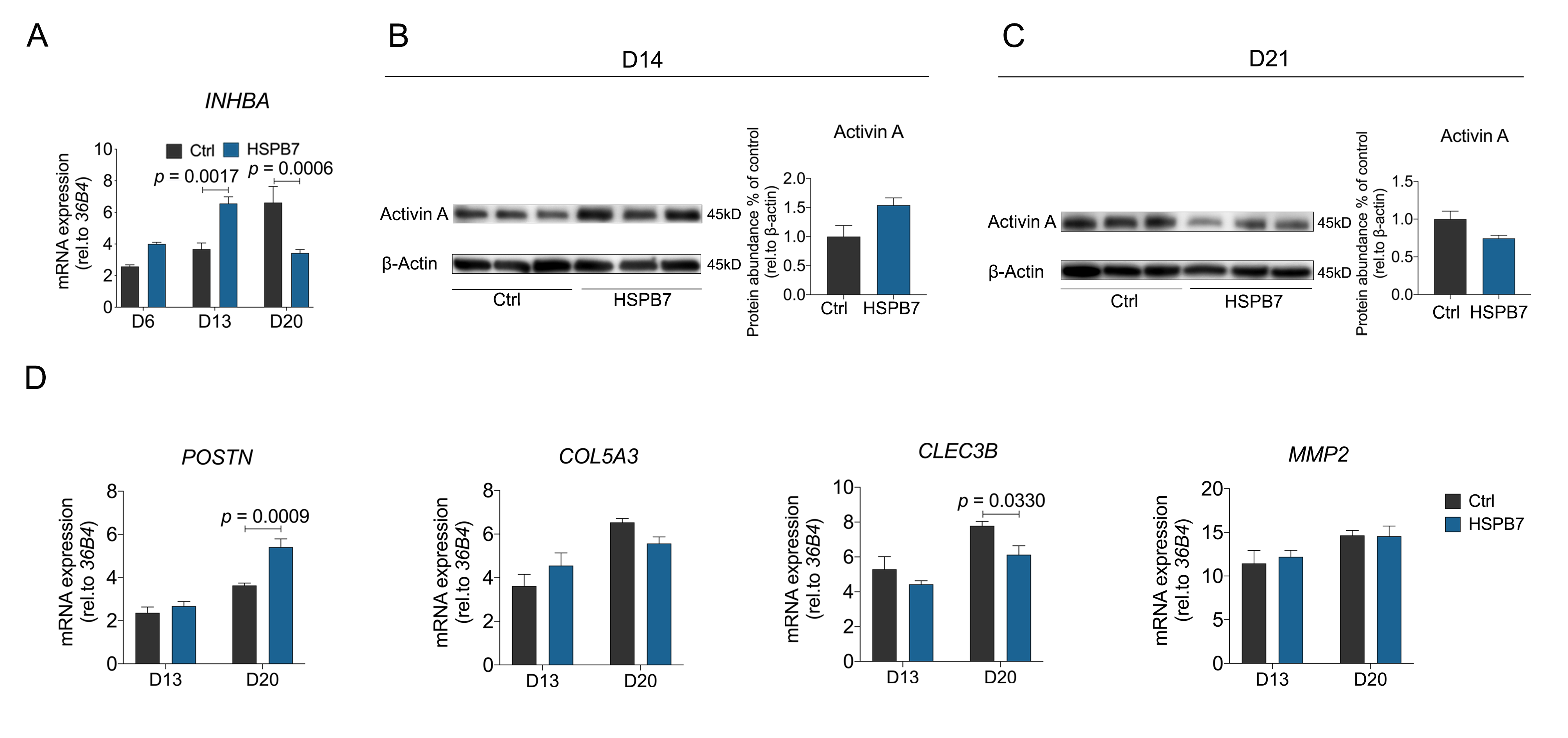

Supplement: Supplementary file 6 — Additional file 6: Fig. S5. Overexpression of HSPB7 affects the expression of extracellular matrix genes. A INHBA mRNA expression was assessed by qRT-PCR following HSPB7 overexpression at multiple time points. B–C Representative images and quantitative expression of activin A were assessed by Western blot at day 14 and day 21 following osteogenic induction. Full-length blots are presented in Additional file 7: Fig. S6. D ECM genes were evaluated by qRT-PCR following HSPB7 overexpression at day 13 and day 20. Data are presented as means ± SEM and analyzed by one-way ANOVA followed by post-hoc testing or two-tailed Student’s t-test. [file 13287_2023_3361_MOESM6_ESM.tif]
