# Supplementary material for: HSPB7 oppositely regulates human mesenchymal stromal cell-derived osteogenesis and adipogenesis
Source: Stem Cell Res Ther. 2023 May 11;14:126. doi: 10.1186/s13287-023-03361-0 (PMC10173662; doi:10.1186/s13287-023-03361-0)
Supplement: Supplementary file 7 — Additional file 7: Fig. S6 Full-length blots. [file 13287_2023_3361_MOESM7_ESM.docx]

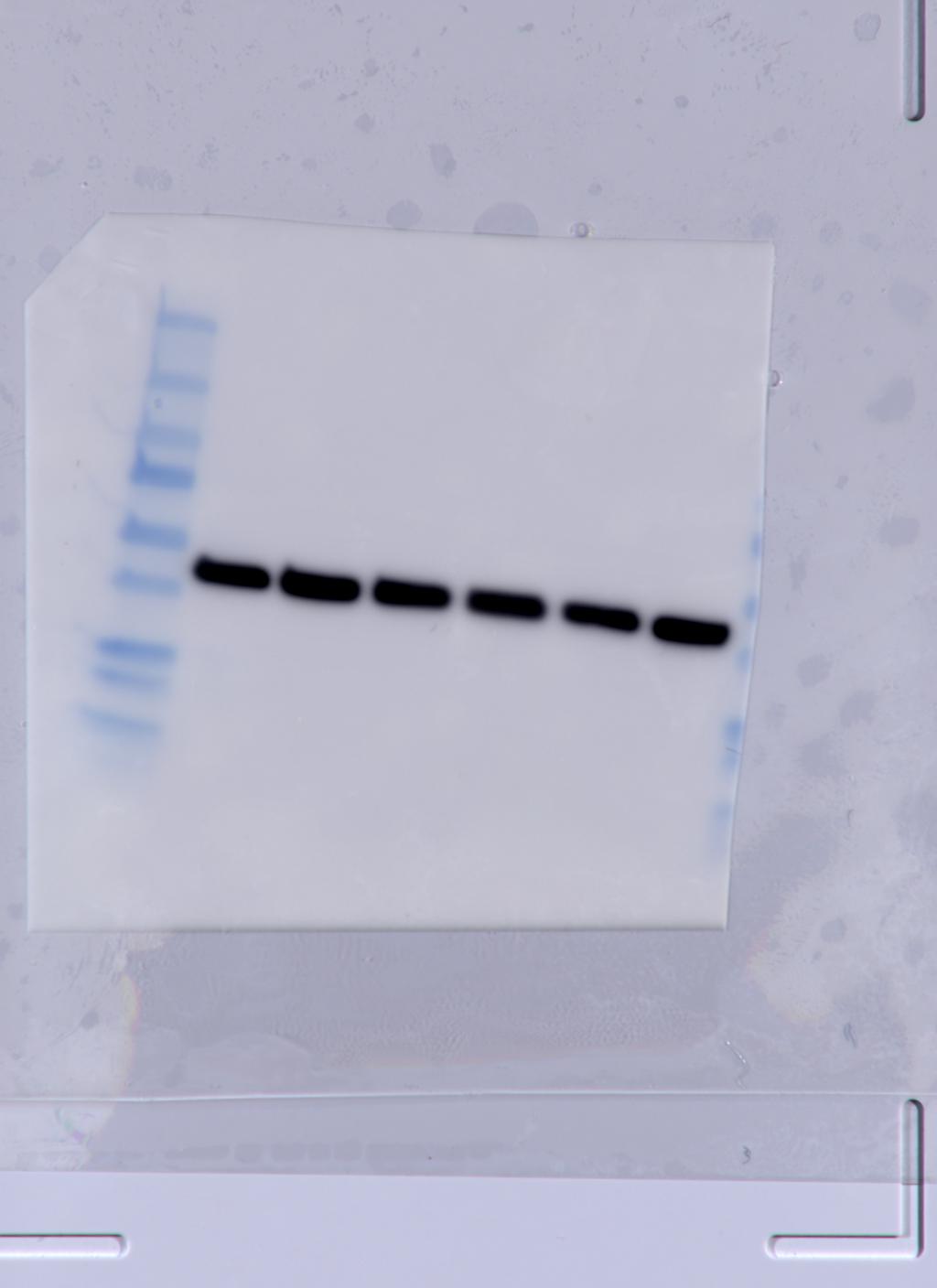


Fig.2B

250 kDa

150 kDa

100 kDa

75 kDa

50 kDa

37 kDa

25 kDa

20 kDa

15 kDa

Actin 45kDa

250 kDa

150 kDa

100 kDa

75 kDa

50 kDa

37 kDa

25 kDa

20 kDa

15 kDa

HSPB7-shRNA2

HSPB7-shRNA1

Ctrl-shRNA

β-Actin 45kDa

HSPB7 19kDa

HSPB7-shRNA1

HSPB7-shRNA2

Ctrl-shRNA

**Supplementary figure 6**


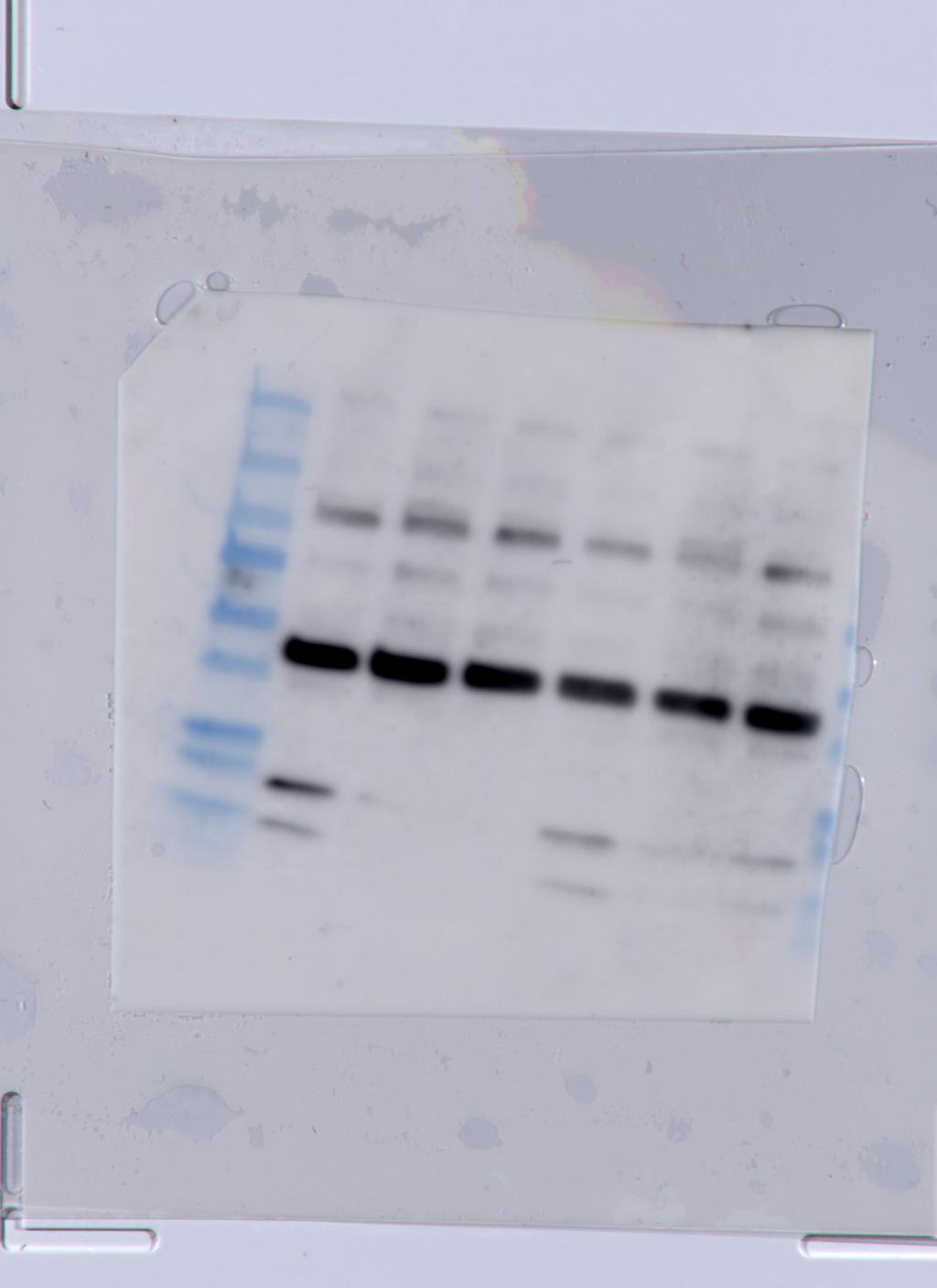


Fig.4F


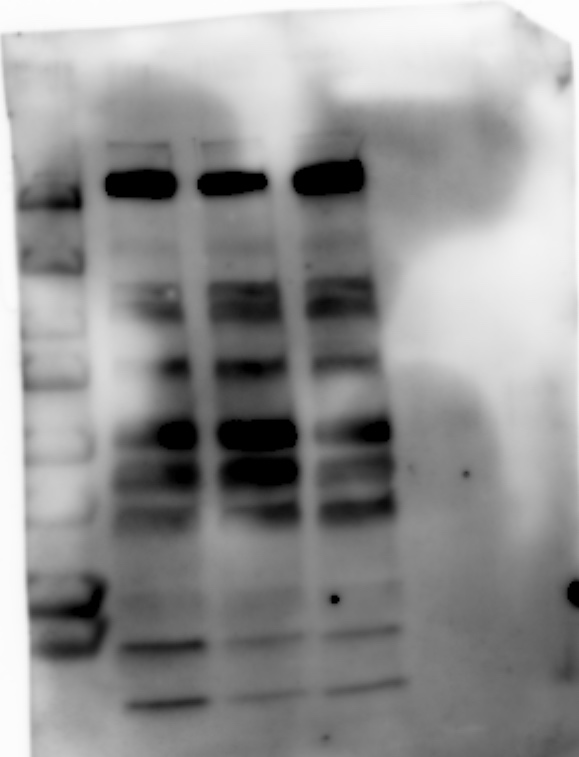

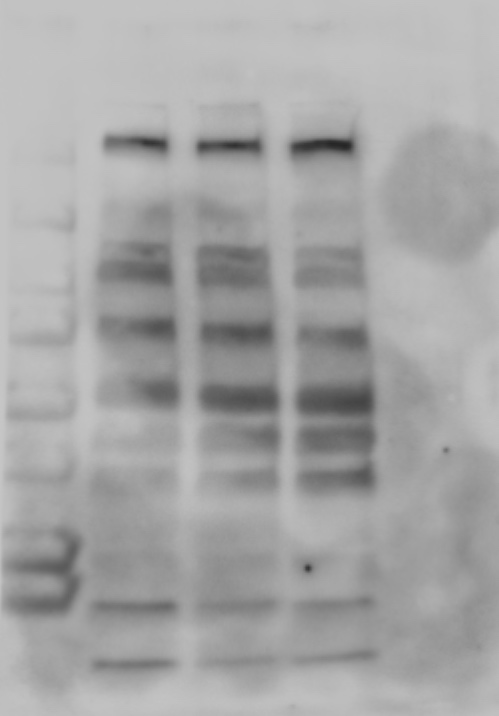


250 kDa

150 kDa

100 kDa

75 kDa

50 kDa

37 kDa

25 kDa

20 kDa

15 kDa

HSPB7 19kDa

HSPB7-shRNA1

HSPB7-shRNA2

Ctrl-shRNA

HSPB7-shRNA2

HSPB7-shRNA1

Ctrl-shRNA

Short exposure

Long exposusre


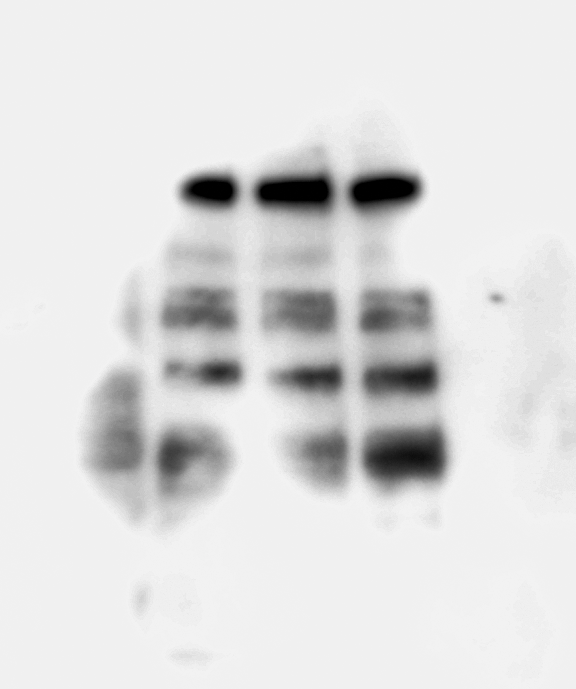

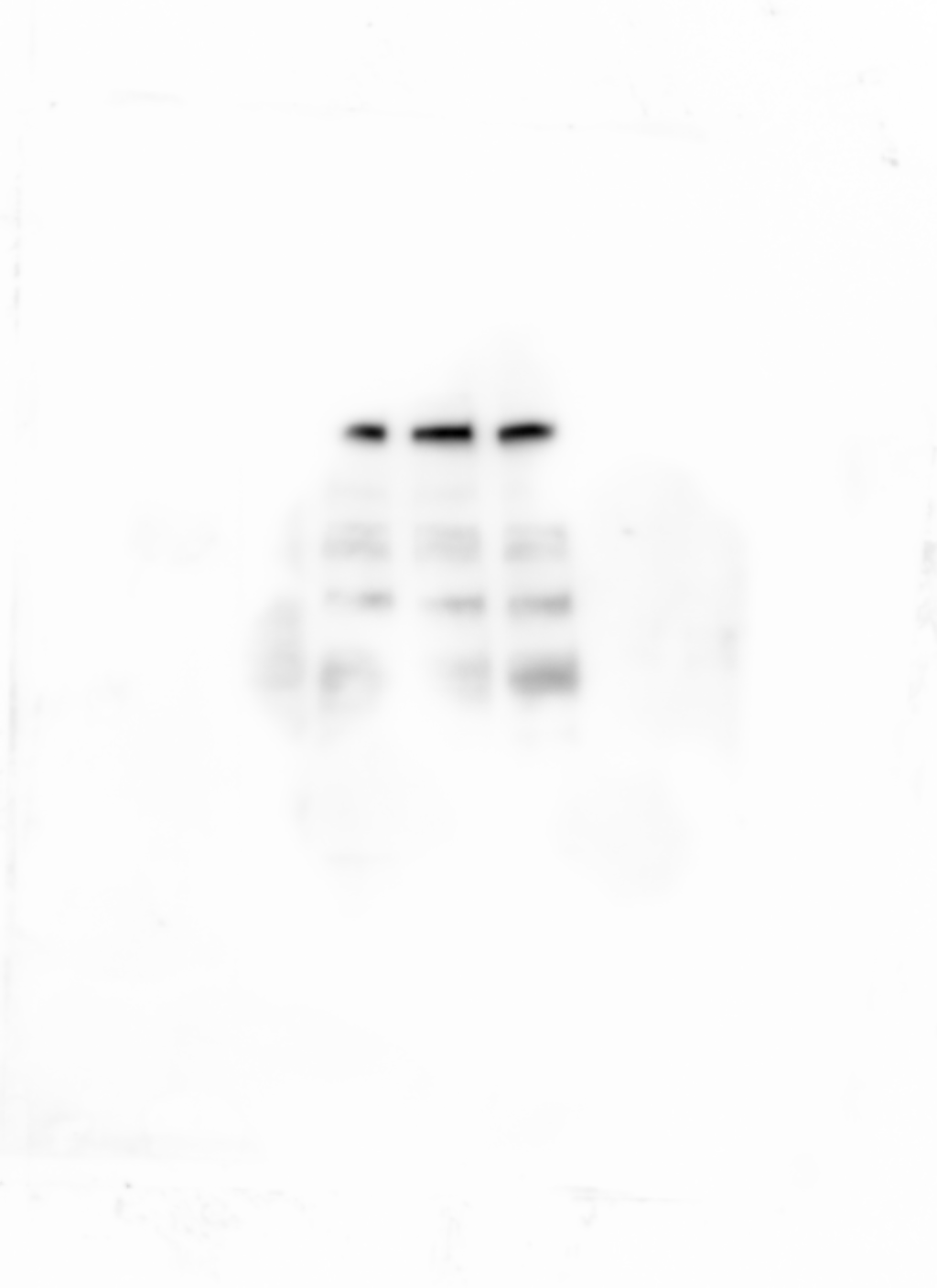


250 kDa

150 kDa

100 kDa

75 kDa

50 kDa

37 kDa

25 kDa

20 kDa

15 kDa

10 kDa

Short exposure

Long exposusre

HSPB7-shRNA2

HSPB7-shRNA1

Ctrl-shRNA

PPARγ 53/57kDa

Ctrl-shRNA

HSPB7-shRNA1

HSPB7-shRNA2


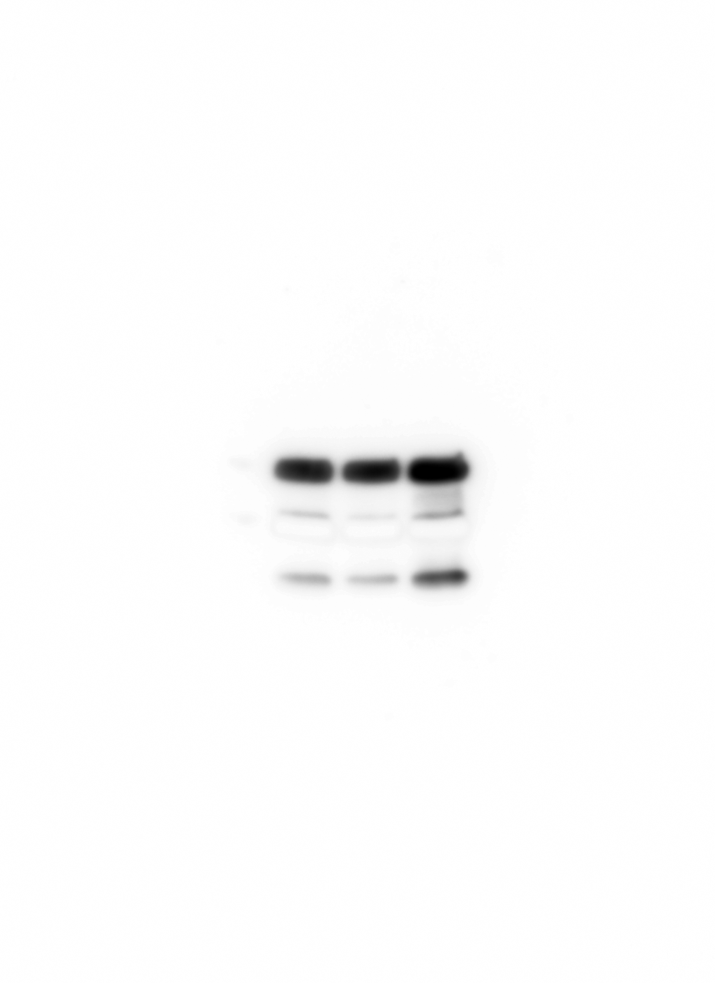


250 kDa

150 kDa

100 kDa

75 kDa

50 kDa

37 kDa

25 kDa

20 kDa

15 kDa

10 kDa

HSPB7-shRNA2

HSPB7-shRNA1

Ctrl-shRNA

CEBPα 42KDa

Perilipin-1 62kDa


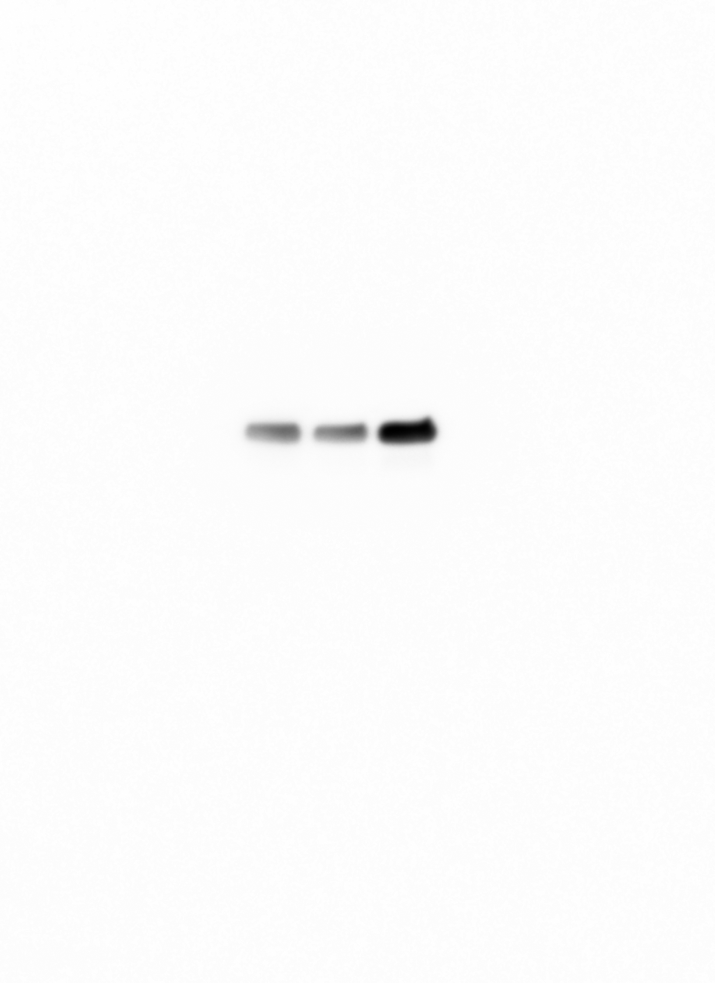


250 kDa

150 kDa

100 kDa

75 kDa

50 kDa

37 kDa

25 kDa

20 kDa

15 kDa

10 kDa

Ctrl-shRNA

HSPB7-shRNA2

HSPB7-shRNA1


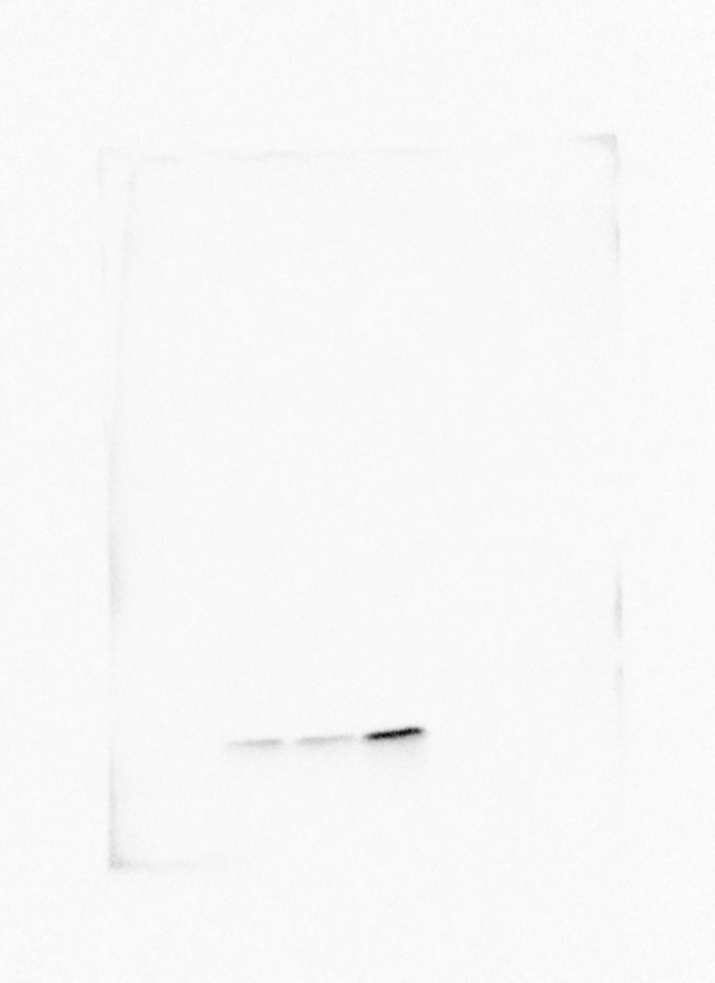


FABP4 15kDa

HSPB7-shRNA2

HSPB7-shRNA1

Ctrl-shRNA

250 kDa

150 kDa

100 kDa

75 kDa

50 kDa

37 kDa

25 kDa

20 kDa

15 kDa

10 kDa


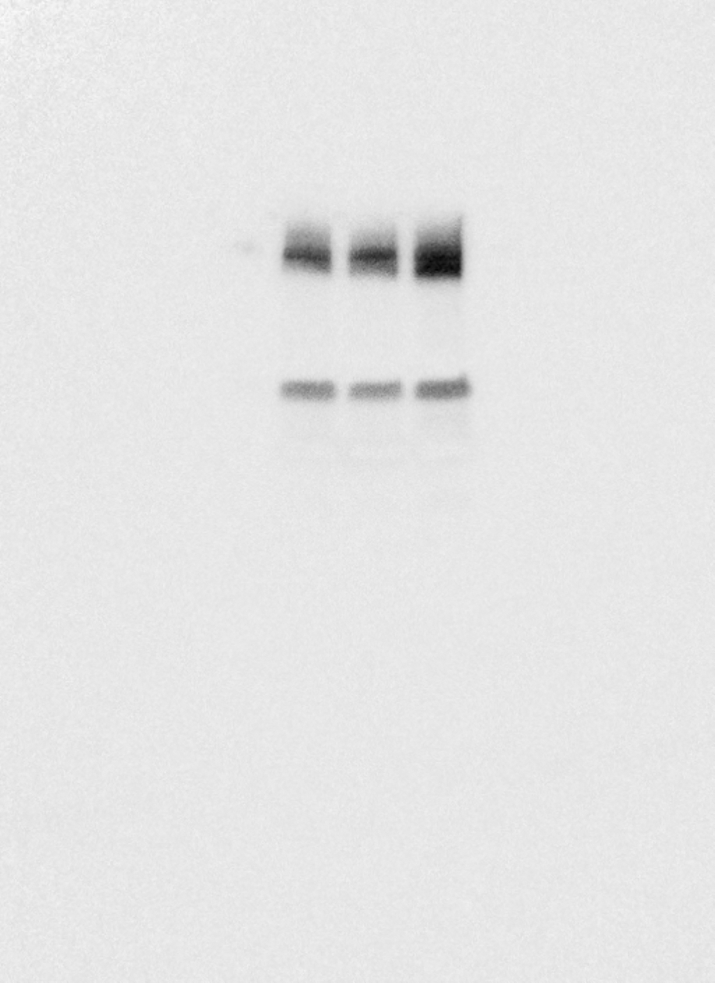


250 kDa

150 kDa

100 kDa

75 kDa

50 kDa

37 kDa

25 kDa

20 kDa

15 kDa

10 kDa

ACC 280 kDa

HSPB7-shRNA2

HSPB7-shRNA1

Ctrl-shRNA


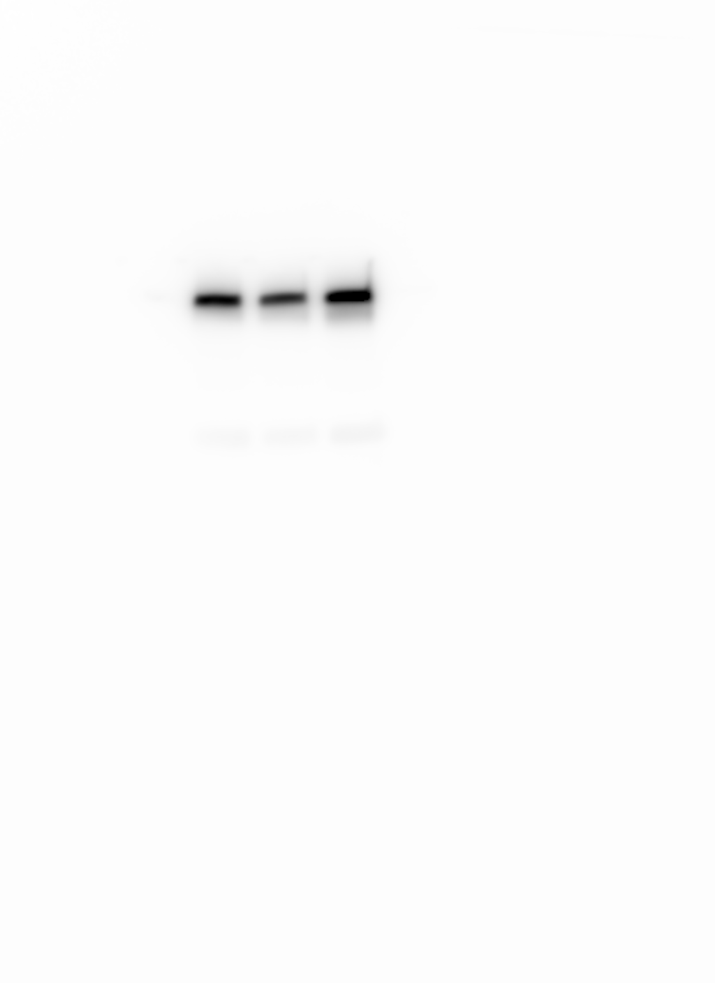

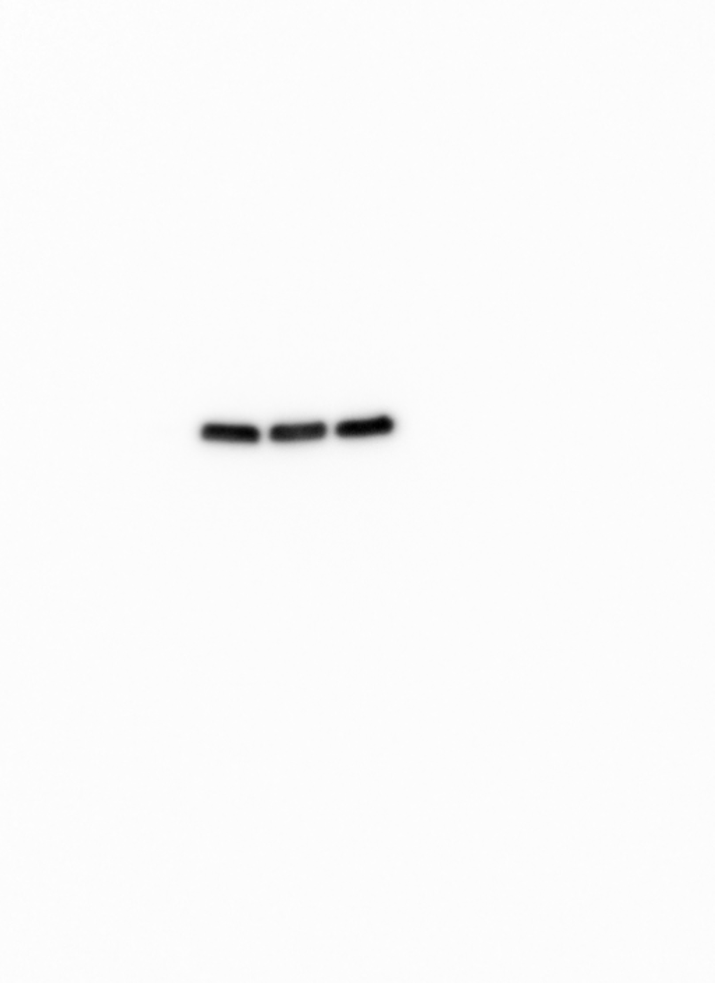


Ctrl-shRNA

HSPB7-shRNA1

HSPB7-shRNA2

β-actin 45kd

250 kDa

150 kDa

100 kDa

75 kDa

50 kDa

37 kDa

25 kDa

20 kDa

15 kDa

10 kDa

250 kDa

150 kDa

100 kDa

75 kDa

50 kDa

37 kDa

25 kDa

20 kDa

15 kDa

10 kDa

FAS 273kDa

HSPB7-shRNA2

HSPB7-shRNA1

Ctrl-shRNA


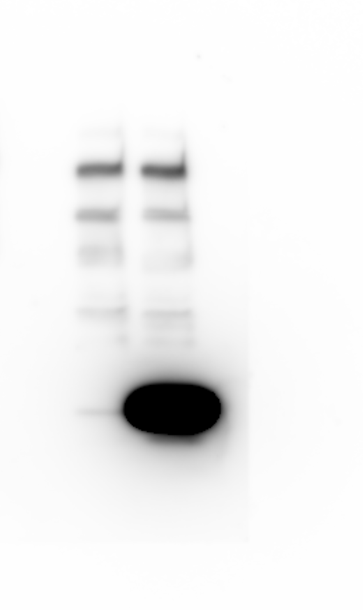

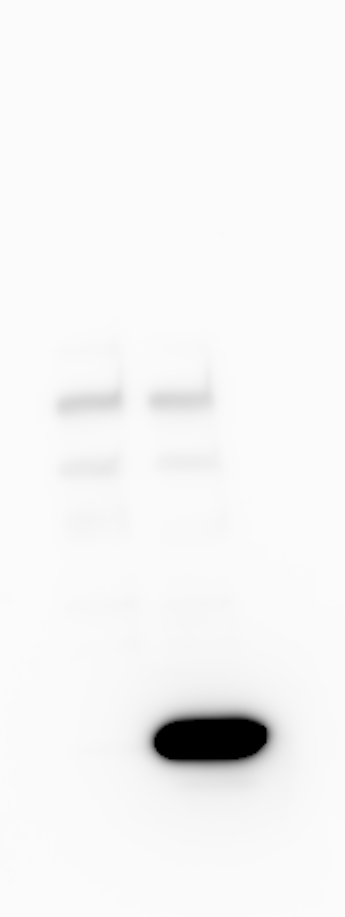


250 kDa

150 kDa

100 kDa

75 kDa

50 kDa

37 kDa

25 kDa

20 kDa

15 kDa

10 kDa

Long exposusre

Short exposure

HSPB7

Ctrl

HSPB7 19kd

Fig.5B

HSPB7

Ctrl


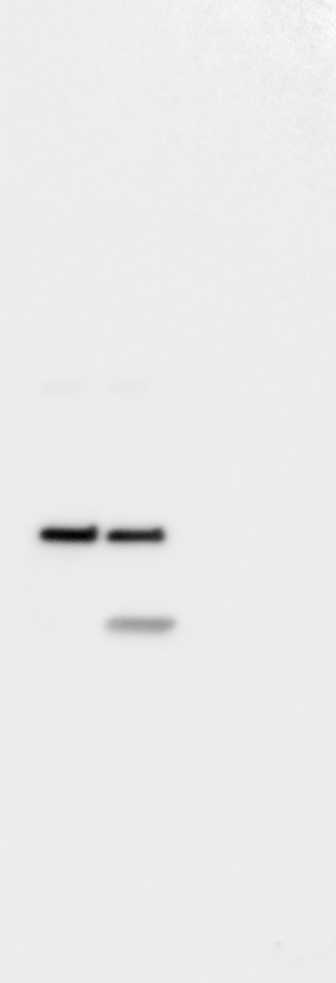


β-actin 45kDa

250 kDa

150 kDa

100 kDa

75 kDa

50 kDa

37 kDa

25 kDa

20 kDa

15 kDa

10 kDa

Ctrl

HSPB7

Fig.6C


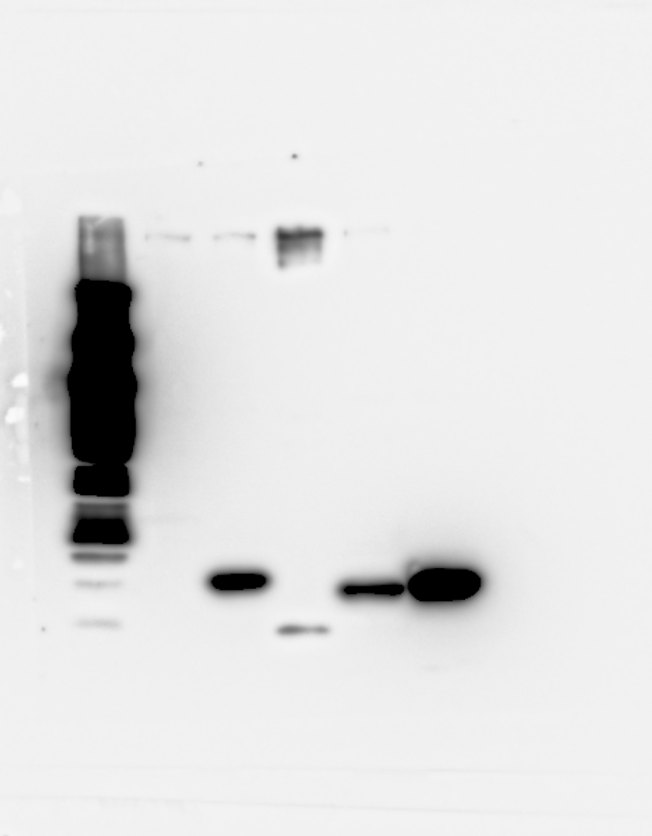

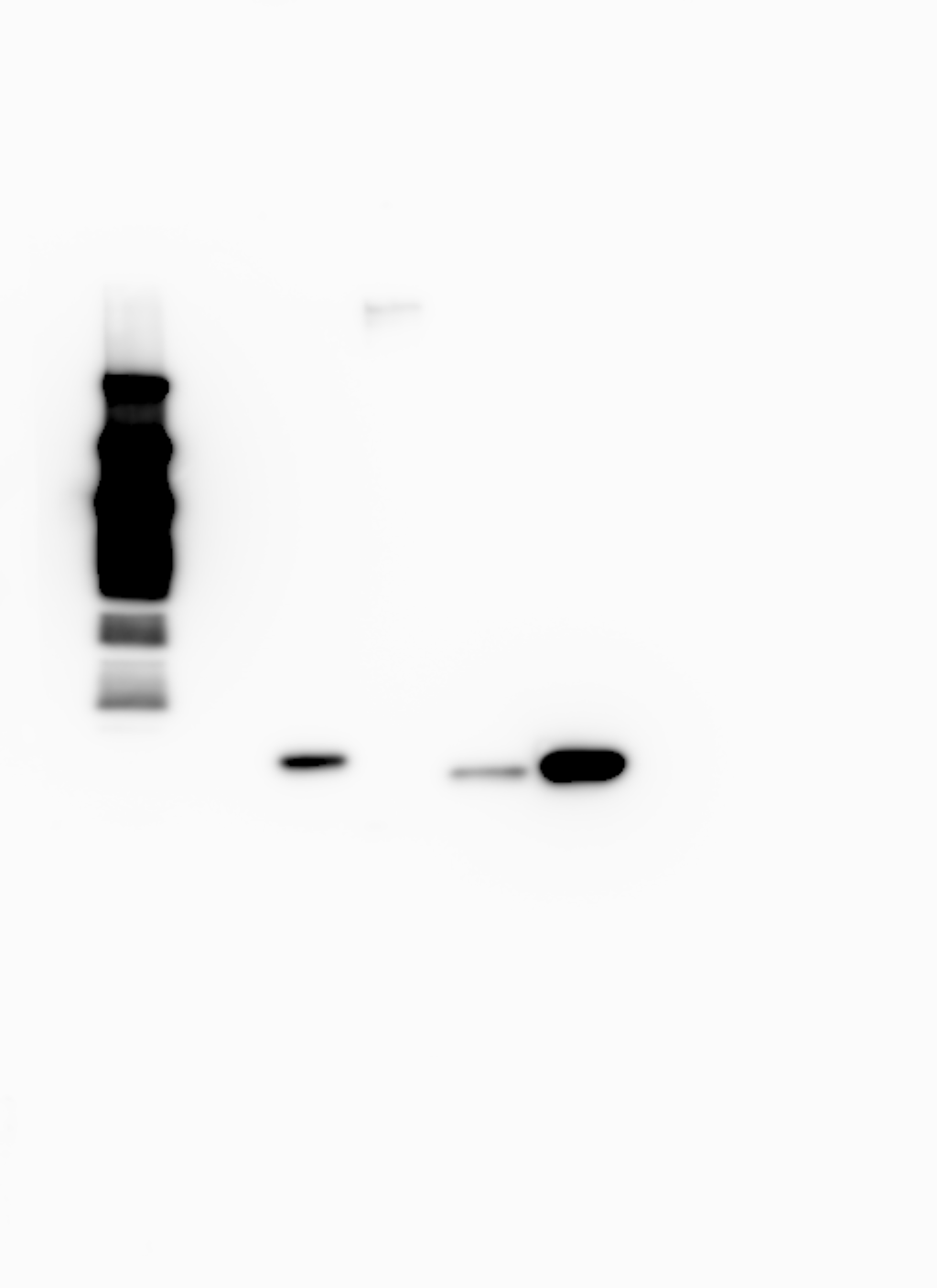


Short exposure

Long exposusre

HSPB7-His

ΔC

ΔSRS

ΔN

FL

Ctrl

His

250 kDa

150 kDa

100 kDa

75 kDa

50 kDa

37 kDa

25 kDa

20 kDa

15 kDa

10 kDa

ΔSRS

ΔC

ΔN

FL

Ctrl

HSPB7-His

FL

ΔN

ΔSRS

ΔC

HSPB7-His

Ctrl


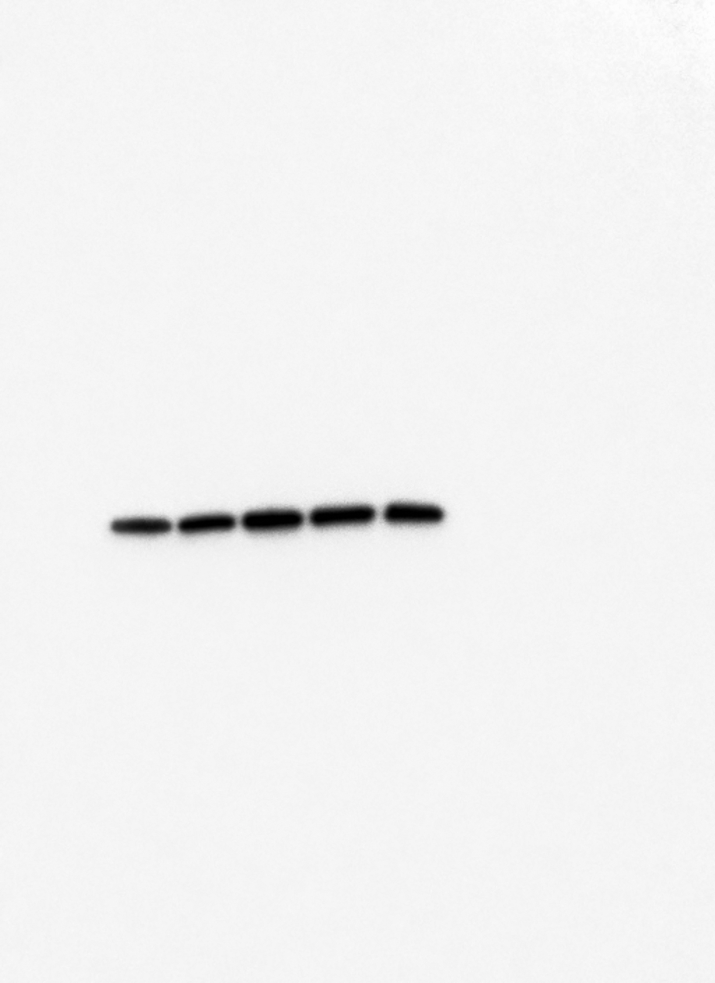


250 kDa

150 kDa

100 kDa

75 kDa

50 kDa

37 kDa

25 kDa

20 kDa

15 kDa

10 kDa

β-actin 45kDa


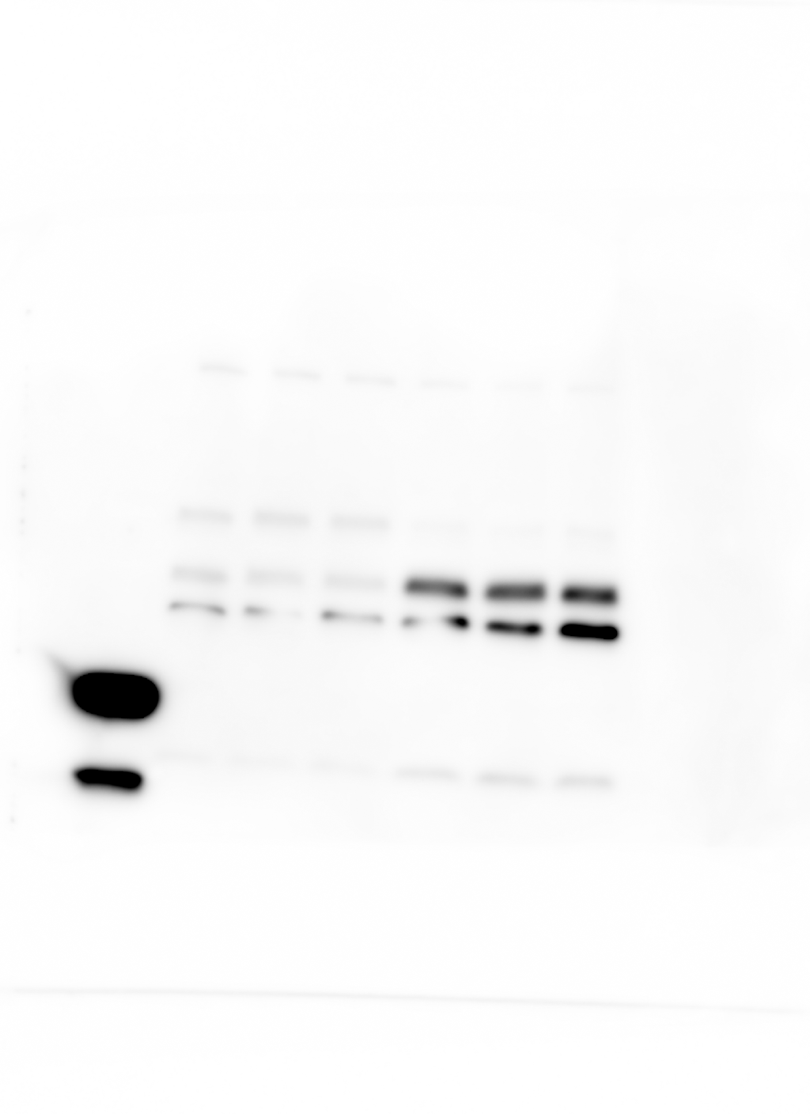

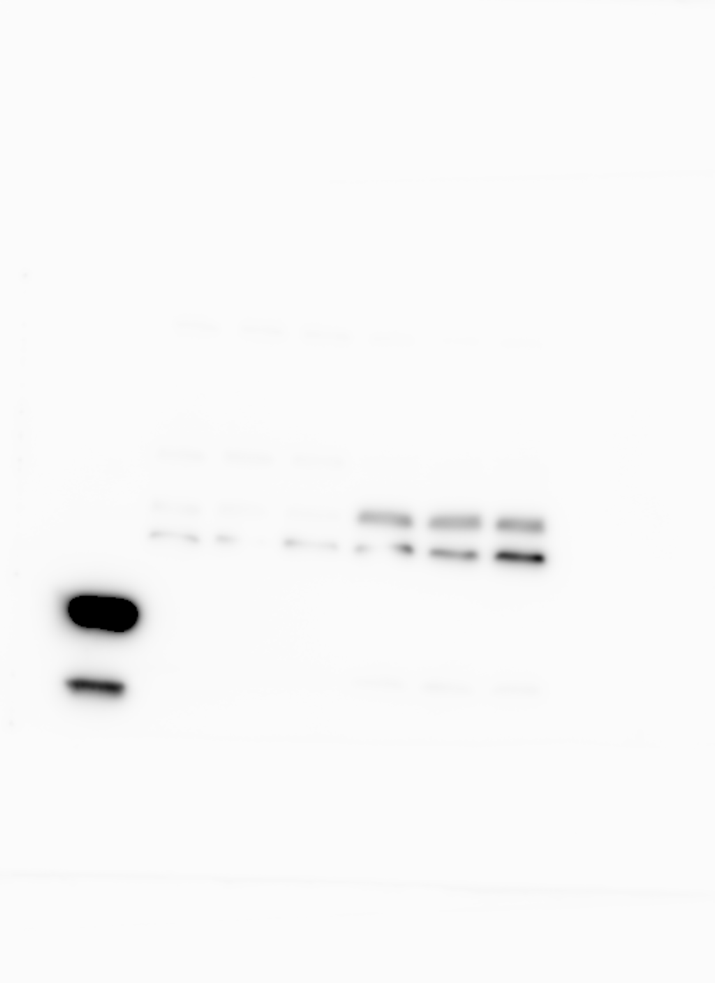


250 kDa

150 kDa

100 kDa

75 kDa

50 kDa

37 kDa

25 kDa

20 kDa

15 kDa

10 kDa

HSPB7-shRNA2

HSPB7-shRNA2

Activin A 45kDa

Ctrl-shRNA

Ctrl-shRNA

Fig.7B


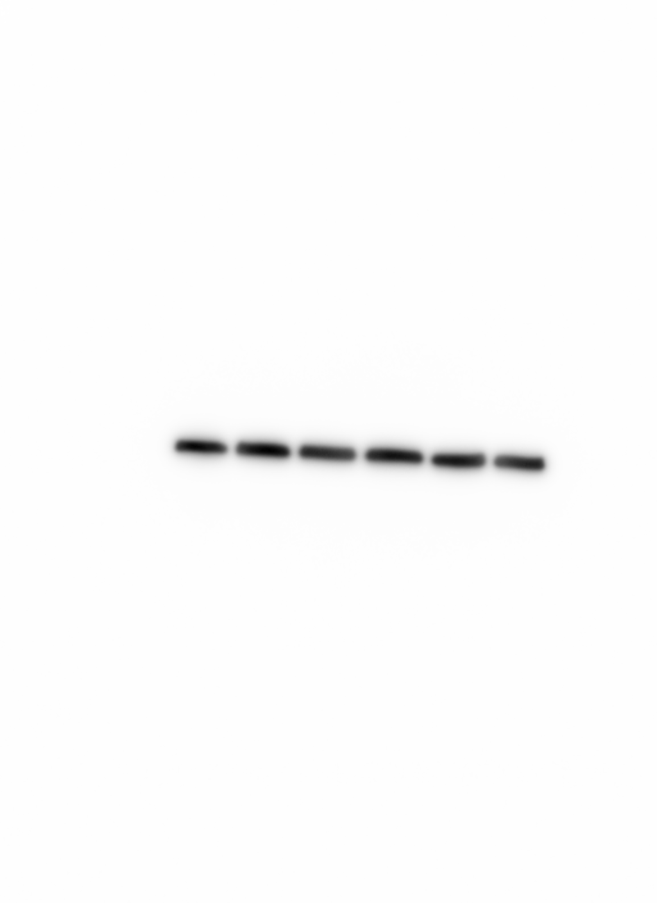


Short exposure

Long exposusre

β-actin 45kDa

Ctrl-shRNA

HSPB7-shRNA2

250 kDa

150 kDa

100 kDa

75 kDa

50 kDa

37 kDa

25 kDa

20 kDa

15 kDa

10 kDa


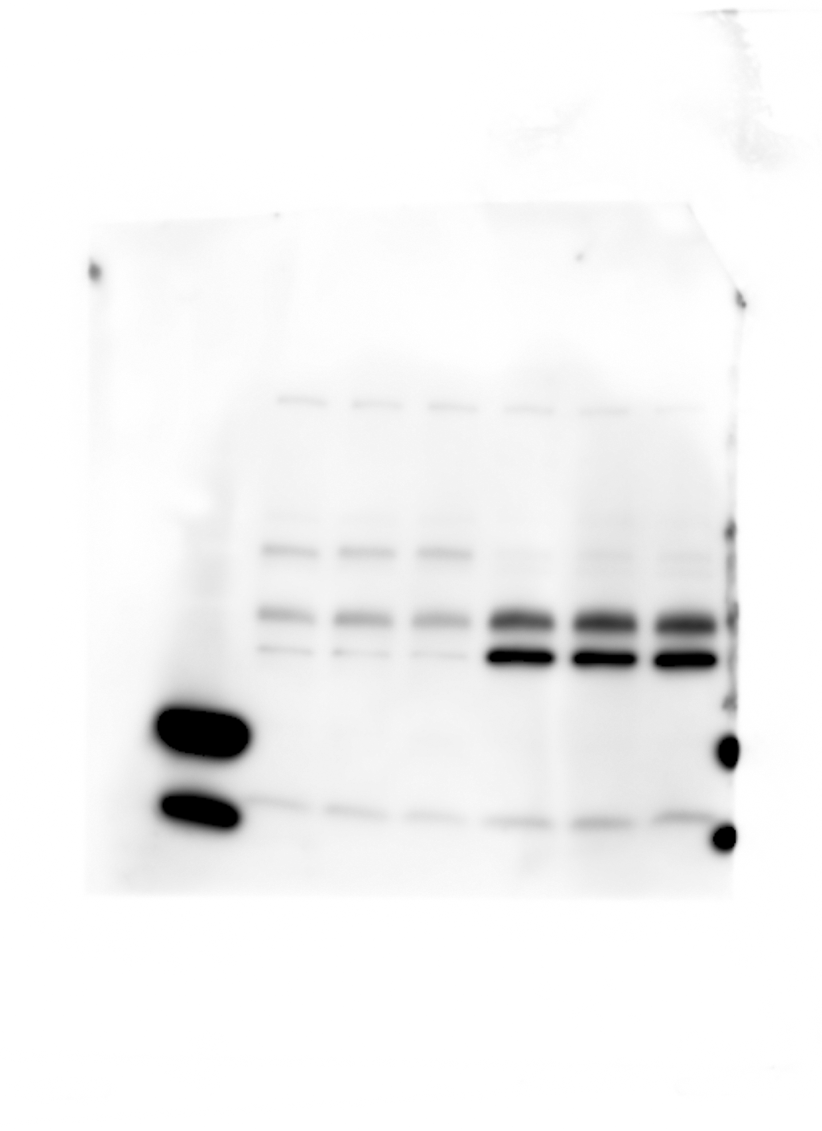

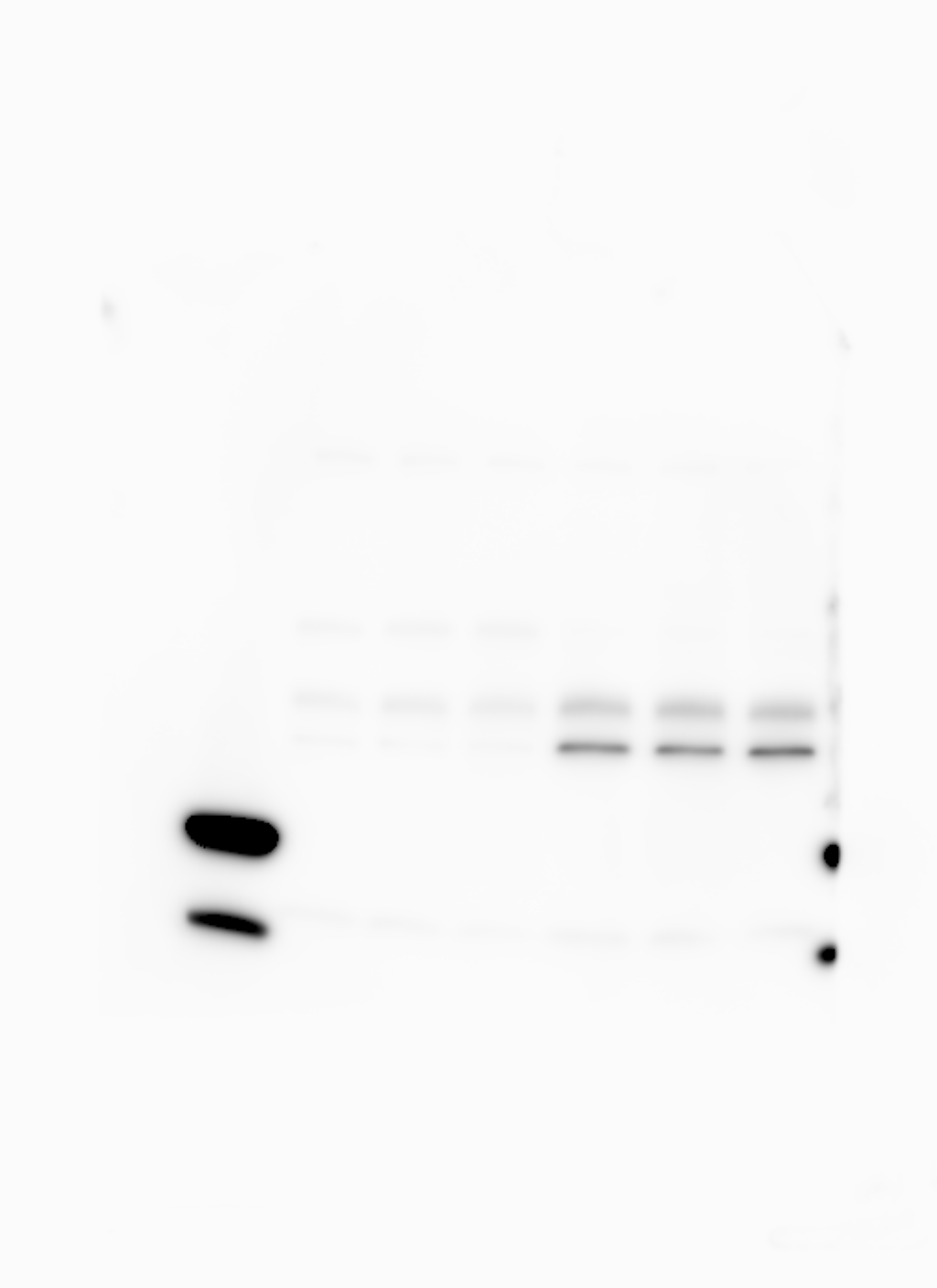


Fig.7C

HSPB7-shRNA2

250 kDa

150 kDa

100 kDa

75 kDa

50 kDa

37 kDa

25 kDa

20 kDa

15 kDa

10 kDa

Activin A 45kDa

Ctrl-shRNA

HSPB7-shRNA2

Ctrl-shRNA


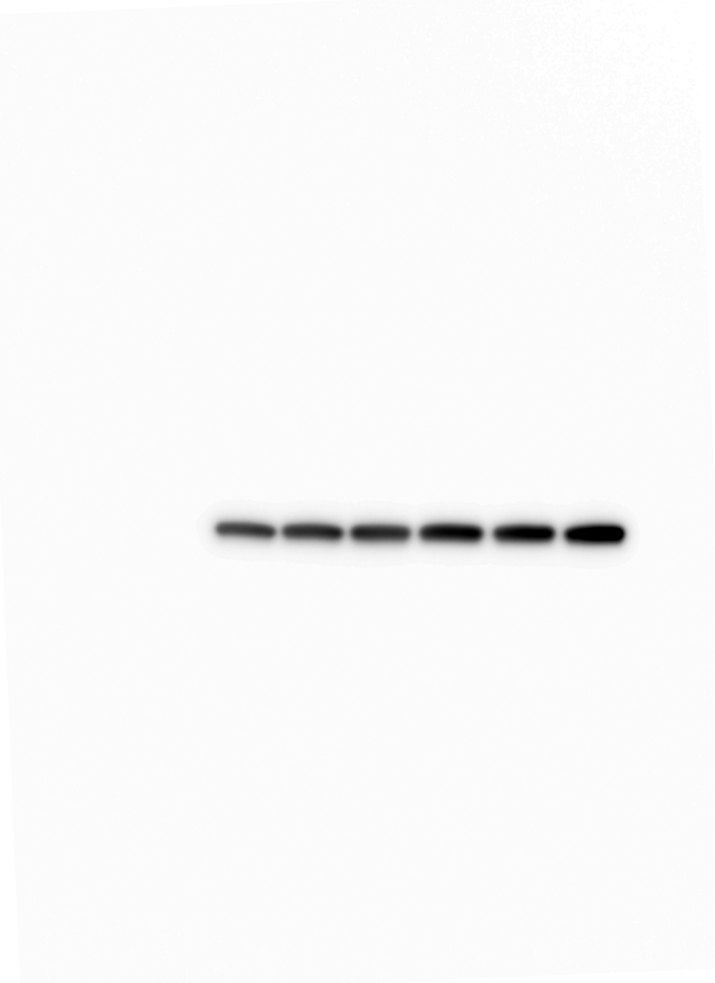


Short exposure

Long exposusre

β-actin 45kDa

Ctrl-shRNA

HSPB7-shRNA2

250 kDa

150 kDa

100 kDa

75 kDa

50 kDa

37 kDa

25 kDa

20 kDa

15 kDa

10 kDa


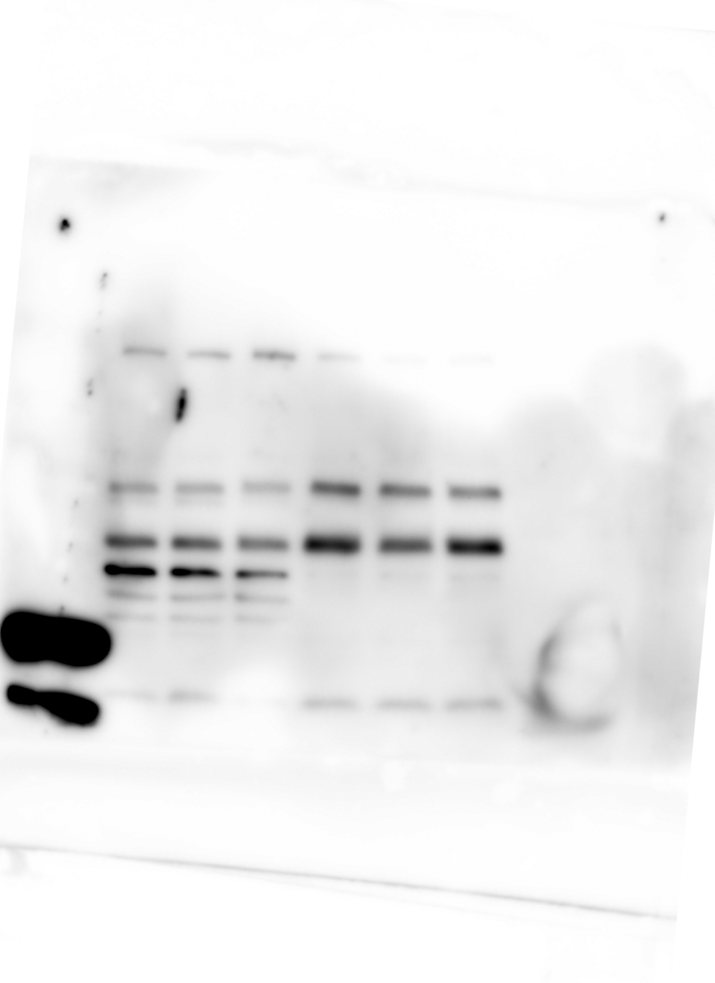


Ctrl

HSPB7

Fig.S5B

Activin A 45kDa

250 kDa

150 kDa

100 kDa

75 kDa

50 kDa

37 kDa

25 kDa

20 kDa

15 kDa

10 kDa


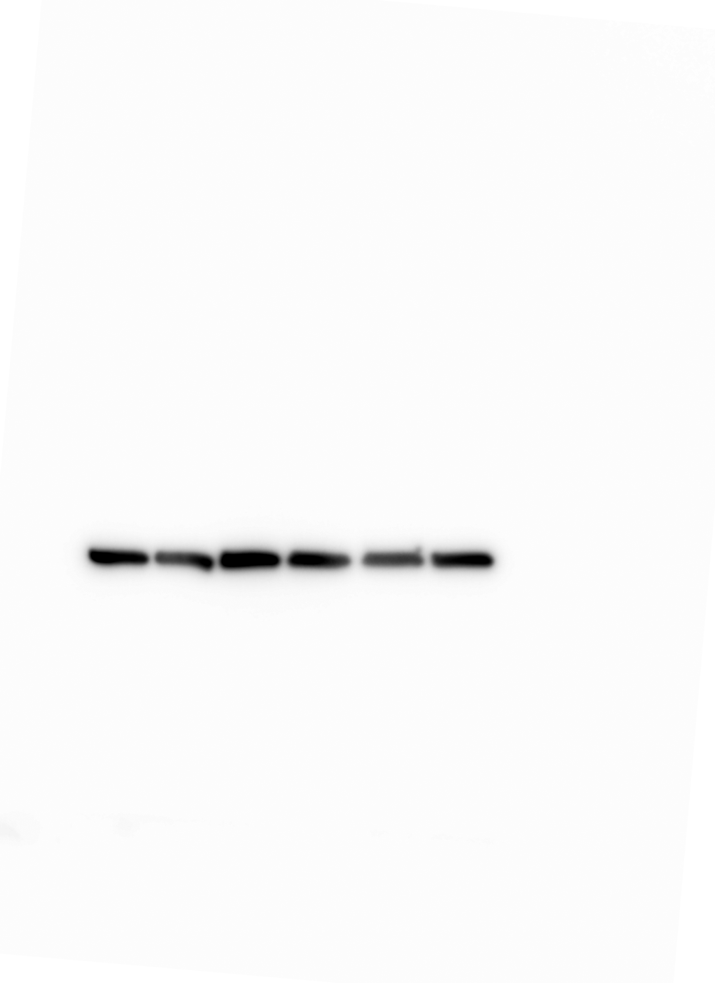

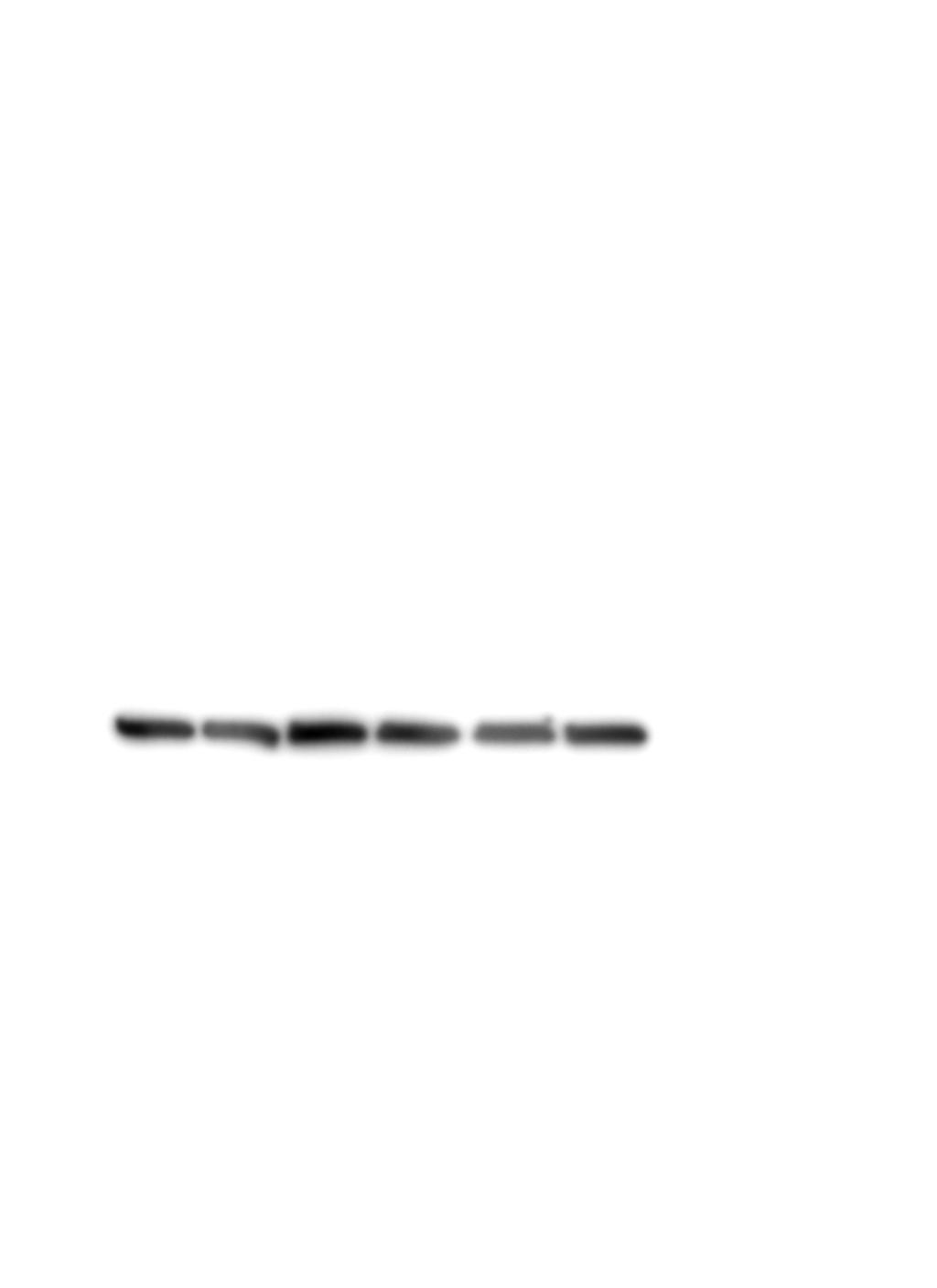


250 kDa

150 kDa

100 kDa

75 kDa

50 kDa

37 kDa

25 kDa

20 kDa

15 kDa

10 kDa

Short exposure

Long exposusre

β-actin 45kDa

HSPB7-shRNA2

Ctrl-shRNA

Ctrl-shRNA

HSPB7-shRNA2


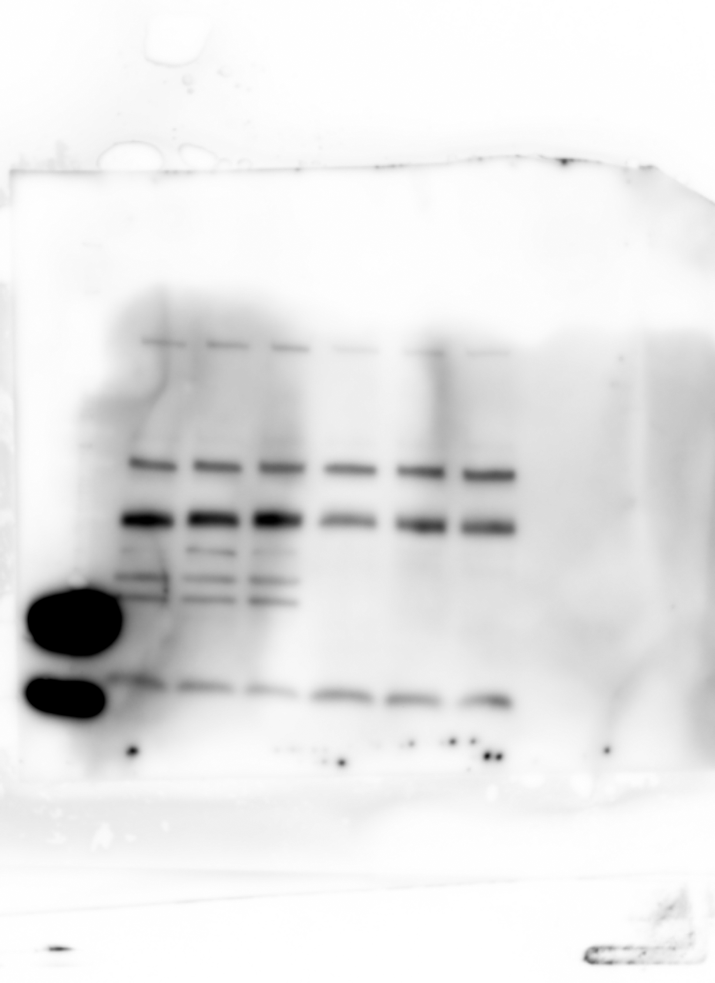


250 kDa

150 kDa

100 kDa

75 kDa

50 kDa

37 kDa

25 kDa

20 kDa

15 kDa

10 kDa

HSPB7

Ctrl

Activin A 45kDa

Fig.S5C


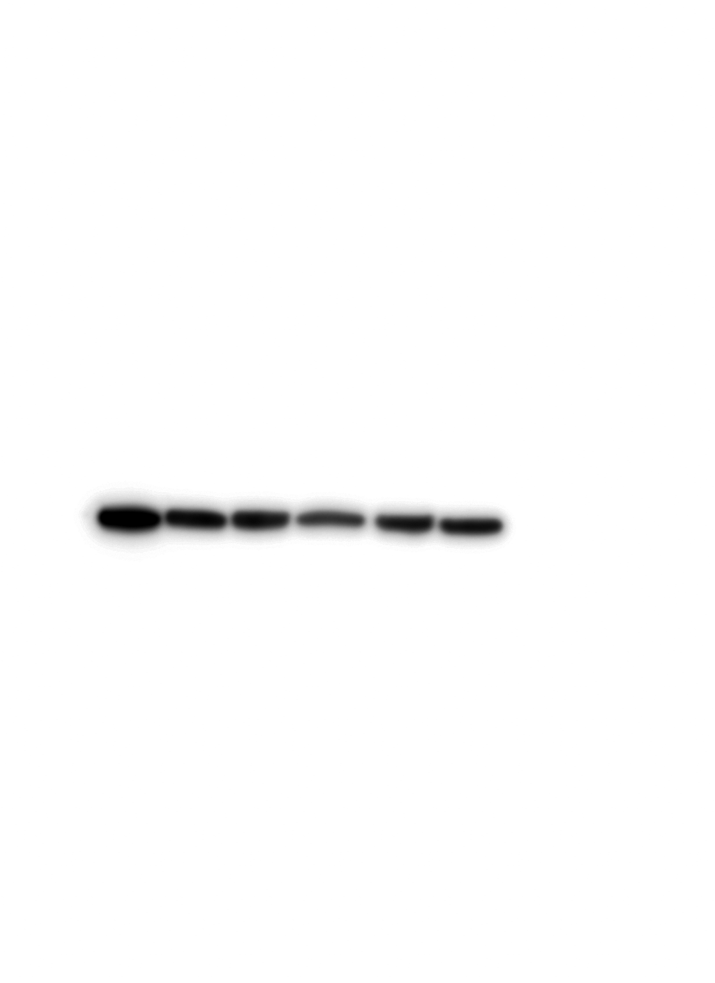

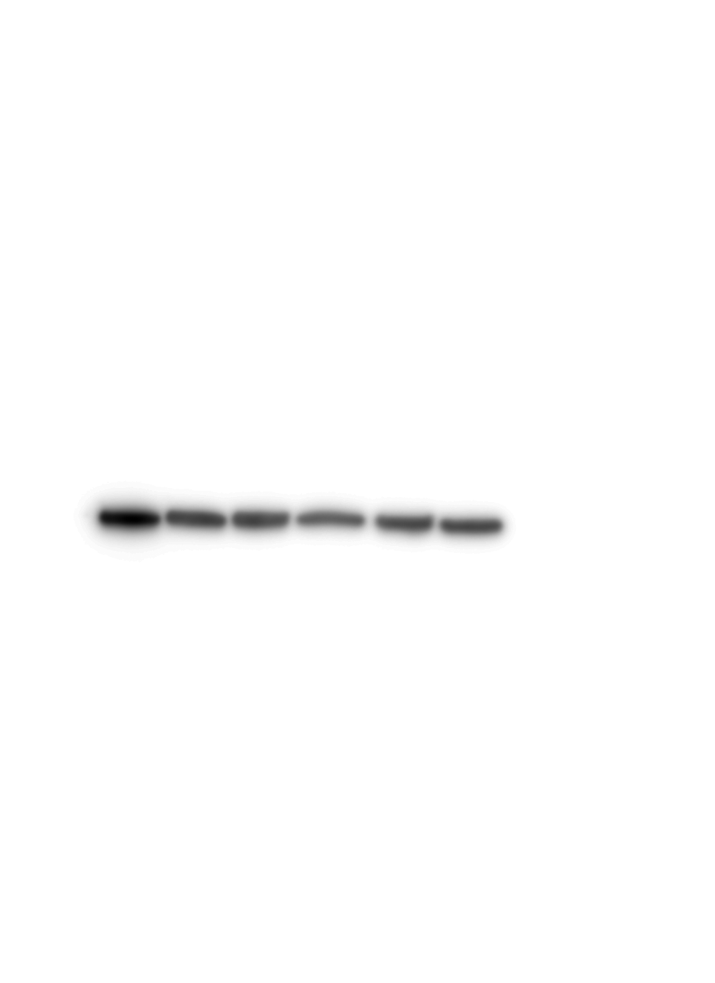


Short exposure

Long exposusre

β-actin 45kDa

HSPB7

Ctrl

HSPB7

Ctrl

250 kDa

150 kDa

100 kDa

75 kDa

50 kDa

37 kDa

25 kDa

20 kDa

15 kDa

10 kDa
